# The Pseudo-Symmetric *N*-benzyl Hydroxyethylamine Core in a New Series of Heteroarylcarboxyamide HIV-1 Pr Inhibitors: Synthesis, Molecular Modeling and Biological Evaluation

**DOI:** 10.3390/biom11111584

**Published:** 2021-10-26

**Authors:** Rosarita D’Orsi, Maria Funicello, Teresa Laurita, Paolo Lupattelli, Federico Berti, Lucia Chiummiento

**Affiliations:** 1 Dipartimento di Scienze, Università della Basilicata, Via Ateneo Lucano 10, 85100 Potenza, Italy; rosarita.dorsi@gmail.com (R.D.); maria.funicello@unibas.it (M.F.); teresalaurita89@gmail.com (T.L.); paolo.lupattelli@unibas.it (P.L.); 2 Dipartimento di Scienze Chimiche e Farmaceutiche, Università di Trieste, Via Licio Giorgieri 1, 34127 Trieste, Italy

**Keywords:** HIV-protease inhibitors, pseudo-symmetric core, heteroaryl carboxyamides, synthesis, biological screening, modeling

## Abstract

Here, we report the synthesis, enzyme inhibition and structure–activity relationship studies of a new potent class of HIV-1 protease inhibitors, which contain a pseudo-symmetric hydroxyethylamine core and heteroarylcarboxyamide moieties. The simple synthetic pathway furnished nine compounds in a few steps with high yields. The compounds were designed taking into account our previous results on other series of inhibitors with different substituents at P’ and P’’ and different ways of linking them to the inhibitor core. Potent inhibitory activity was obtained with nanomolar IC_50_ values measured with a standard fluorimetric test in 100 mM MES buffer, pH 5.5, containing 400 mM NaCl, 1 mM EDTA, 1 mM DTT and 1 mg/ml BSA. Compounds **9a**–**c**, containing the indole ring in P1, exhibited an HIV-1 protease inhibitory activity more powerful than darunavir in the same assay. To obtain molecular insight into the binding properties of these compounds, docking analysis was performed, and their binding properties were also compared.

## 1. Introduction

The AIDS epidemic is still one of the most challenging problems [1], although great efforts are made for the discovery of new drugs for its treatment. Among many strategies to combat the disease, antiretroviral therapy (ART) containing at least one HIV-1 protease inhibitor (PIs) is considered the most effective treatment [2,3,4,5]. When a protease inhibitor binds to the active site, it prevents the cleavage of nascent viral proteins, thereby halting viral replication [6]. The synthesis of compounds able to block the action of the HIV protease, the enzyme which plays a key role in maintaining infectivity, is currently a huge aim.

Nowadays, nine FDA-approved PIs are available on the market, but due to the rapid genomic evolution of HIV, an inevitable consequence in the treatment of the infection has been the rise of drug resistance, and therefore, the dramatic reduction of the marketed inhibitors efficiency [7,8].

Thus, the emergence of highly mutated viral strains cross-resistant to antivirals, the occurrence of various side effects and the high cost of ART prompted scientists to seek novel PIs, preferably with alternative frameworks. 

Notably, the introduction of heterocyclic moieties in a bioactive molecule can have important effects on physicochemical and pharmacological properties [9]. This strategy has been widely adopted in medicinal chemistry for the design of new drugs, because of their chemical stability and structural rigidity, less entropic energy was lost upon binding. In our experience of the synthesis of highly functionalized small molecules with aryl and heteroaryl structures [10,11,12], we evidenced the crucial effect of the presence of heterocyclic moiety in PIs structure, either in the core of the inhibitors [13,14,15] or in the P2 position [16,17,18].

**Figure 1:**
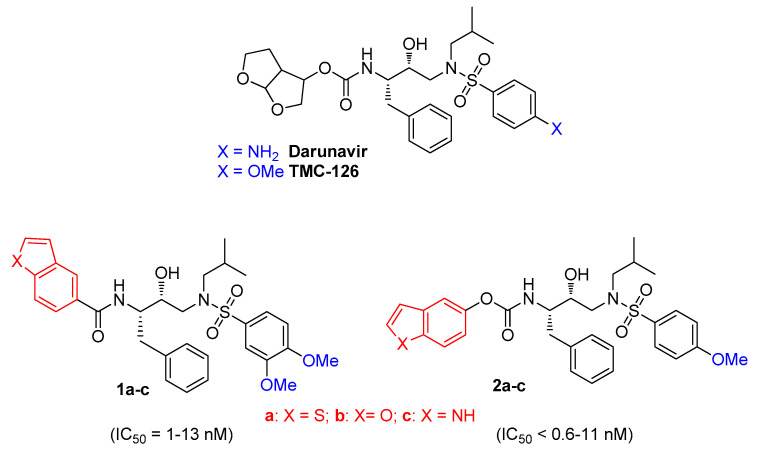
Commercial and not HIV-protease inhibitors.

Inspired by the success of darunavir, an FDA-approved drug for the treatment of HIV, and its analog TMC-126, containing a bis-tetrahydrofuran heterocyclic system as P2 ligand (Figure 1), our aim was focused on designing inhibitors containing heteroaryl moieties that specifically target and maximize interactions with the backbone. Both extensive hydrogen bonding and hydrophobic interactions with enzyme subsites can limit the protease’s ability to acquire drug resistance as the geometry of the catalytic site must be conserved to maintain functionality [19,20,21].

Recently the preparation and the activity, in vitro and in mammalian cells, of new HIV protease inhibitors, compounds 1 and 2, were reported by our group (Figure 1) [22,23].

They were designed having heterocycle as the P2 ligand linked by a carboxyamidic or carbammic moiety to the core, with or without the benzyl group, and a 3,4-dimethoxyphenylsulfonyl-*N*-isobutylamide [22] or a 4-methoxyphenylsulfonyl-*N*-isobutylamide [23] as the P2’ ligand. Compounds with benzyl in the core showed in vitro activity against native protease with IC_50_ values in the range of <0.6–13 nM.

**Figure 2:**
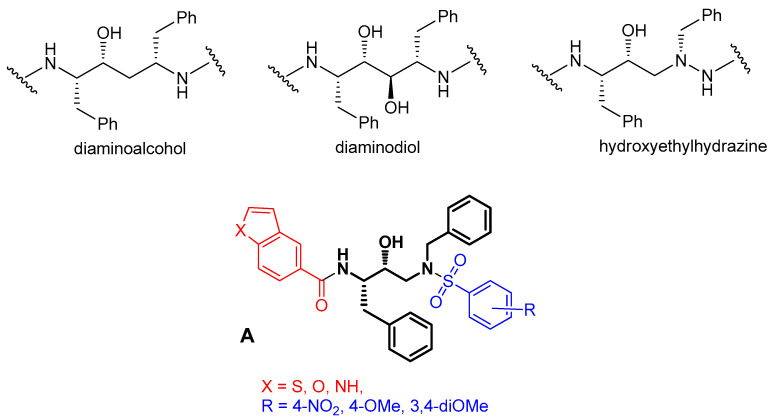
Examples of pseudo-symmetrical core of HIV-protease inhibitors.

As HIV protease has been shown to exist as a C2-symmetric homodimer in its active form, several dipeptide isosteres, such as diaminoalcohol, diaminodiol and hydroxyethylhydrazine, have also been employed in the development of pseudo-symmetric inhibitors (that is, inhibitors that are lacking the same C2 symmetry of the enzyme, but bear the same group at P1 and P1’) [24] (Figure 2).

In order to obtain a pseudo-symmetric hydroxyethylamine core*,* the isobutyl portion present in the structure of compounds **1** and **2** (Figure 1) was replaced with a benzyl group. Therefore, a library of compounds containing different heteroarenes and sulfonamide portions was prepared. The general structure of these newly synthesized compounds is reported as **A** in Figure 2. The structure takes into account the outcomes of our previous evaluations and the indications obtained in the models developed and described in refs [13,14,16,17,18,22,23]. 

The effects on the inhibitory activity of the heteroatom (S, O, N) in the heteroarylcarboxamidic portion and the electronic properties of the substituents on the sulfonamidic moiety were also evaluated. The novel inhibitors were tested in vitro on HIV-1 protease with a standard fluorometric assay, and their activities were compared with that of darunavir. 

## 2. Materials and Methods

### 2.1. Chemistry

Preparative chromatography was carried out on Merck silica gel (0.063–0.200 mm particle size) by progressive elution with opportune solvent mixtures. ^1^H and ^13^C NMR spectra were normally carried out in CDCl_3_ solutions on a VARIAN INOVA 500 MHz or Bruker 400 MHz and referenced to CDCl_3_. Mass spectra were obtained with a Hewlett-Packard 5971 mass-selective detector on a Hewlett–Packard 5890 gas chromatograph ((OV-1 capillary column between 70 and 250 °C (20 °C min^−1^)). The optical purity was evaluated by using a polarimeter JASCO Mod Dip-370. CH_2_Cl_2_ was dried by distillation over anhydrous CaCl_2_ in an inert atmosphere. Dry THF and DMF were commercially available.

***tert*-butyl (2*S*,3*R*)-4-(benzylamino)-3-hydroxy-1-phenylbutan-2-ylcarbamate (4).** Compound **4** was prepared from a solution of (2***S***,3***S***)-1,2-epoxy-3-(Boc-amino)-4-phenylbutane (1.5 mmol) and benzylamine (1.5 mmol) in ***i***-PrOH (10 mL) that was stirred under reflux for 16 h. The reaction mixture was rotary evaporated, and the crude product was purified by recrystallization in methanol/water (7:3). Compound **4** was obtained as a white solid, yield 88%. ^1^H and ^13^C NMR spectra were consistent with the data in the literature [25].

**General procedure for the preparation of *tert*-butyl ((2*S*,3*R*)-*n*-[4-(*n*-benzyl-4-R-phenylsulfonamido)-3-hydroxy-1-phenylbutan-2-yl)carbamates (5).** To a stirred solution of aminoalcohol 4 (0.78 mmol) in anhydrous CH_2_Cl_2_ (20 mL), Et_3_N (2.02 mmol) and arylsulfonyl chloride (0.93 mmol) were added at room temperature and under an Ar atmosphere. After 24 h, the reaction was quenched with 5% aqueous H_2_SO_4_ solution. The organic layer was washed, adding saturated aqueous NaHCO_3_ solution and brine. The organic phases collected were dried over Na_2_SO_4,_ filtered and concentrated in vacuo. The crude product was purified by column chromatography on silica gel.

**(1*S*,2*R*)-{1-Benzyl-2-hydroxy-3-[*n*-benzyl-(4-nitrobenzenesulfonyl)-amino]-propyl}-carbamic acid *tert*-butyl ester (5a).** Compound **5a** was isolated as a white solid (CH_2_Cl_2_/EtOAc 98/2), yield 94%. $\left[\alpha \right]{\;}_{D}^{20}=$ +6.3° (c: 1.0, CHCl_3_). ^1^H NMR (400 MHz, DMSO-d_6_): δ 8.37 (d, *J* = 9.0 Hz, 2H), 8.10 (d, *J* = 9.0, 2H), 7.33 (m, 5H), 7.21 (m, 2H), 7.13 (m, 3H), 6.63 (d, *J* = 9.0 Hz, 1H), 5.00 (d, *J* = 6.5, 1H), 4.68 (d, *J* = 15.5 Hz, 1H), 4.44 (d, *J* = 15.5 Hz, 1H), 3.46 (m, 2H), 3.35 (m, 1H), 3.10 (dd, *J*_1_ = 15.0 Hz, *J*_2_ = 9.0 Hz, 1H), 2.89 (dd, *J*_1_ = 13.7 Hz, *J*_2_ = 3.0 Hz, 1H), 2,42 (dd, *J*_1_ = 13.5 Hz, *J*_2_ = 11.0 Hz, 1H), 1.20 (s, 9H). ^13^C{1H} NMR (100 MHz, DMSO-d_6_): δ 155.2, 149.5, 146.1, 139.3, 136.1, 129.0, 128.5, 128.4, 128.1, 127.8, 127.5, 125.6, 124.3, 77.5, 71.2, 54.9, 50.9, 50.2, 35.2, 28.1. Anal. Calcd for C_28_H_33_N_3_O_7_S: C, 60.52; H, 5.99; N, 7.56; S, 5.77. Found: C, 60.54; H, 5.93; N, 7.50; S,5.75.

**(1*S*,2*R*)-{1-Benzyl-2-hydroxy-3-[*n*-benzyl-(4-methoxybenzenesulfonyl)-amino]-propyl}-carbamic acid *tert*-butyl ester (5b).** Compound **5b** was obtained as a white solid (CH_2_Cl_2_/EtOAc 95/5), yield 85 %. $\left[\alpha \right]{\;}_{D}^{20}=$ +5.4° (c: 1.0, CHCl_3_). ^1^H NMR (400 MHz, DMSO-d_6_): δ 7.78 (d, *J* = 8.5 Hz, 2H), 7.28 (m, 8H), 7.16–7.08 (m, 4H), 6.60 (d, *J* = 8.8 Hz, 1H), 4.94 (d, *J* = 6.0 Hz, 1H), 4.50 (d, *J* = 15.6 Hz, 1H), 4.37 (d, *J* = 15.6, 1H), 3.85 (s, 3H), 3.48 (m, 2H), 3.35 (m, 1H), 2.93 (m, 2H), 2.45 (dd, *J* = 13.8 Hz, *J* = 10.4 Hz, 1H), 1.22 (s, 9H). ^13^C{1H} NMR (100 MHz, DMSO-d_6_): δ 155.2, 139.5, 136.6, 131.6, 129.2, 129.1, 128.2, 128.0, 127.8, 127.2, 125.6, 114.3, 77.4, 72.0, 54.9, 55.6, 54.8, 51.4, 50.7, 35.2, 28.1. Anal. Calcd for C_29_H_36_N_2_O_6_S: C, 64.42; H, 6.71; N, 5.18; S, 5.93. Found: C, 64.49; H, 6.76; N, 5.23; S, 5.88.

**(1*S*,2*R*)-{1-Benzyl-2-hydroxy-3-[*n*-Benzyl-(3,4-dimethoxybenzenesulfonyl)-amino]-propyl}-carbamic acid *tert*-butyl ester (5c).** Compound **5c** was obtained as a white solid (CH_2_Cl_2_/EtOAc 95/5), yield 90 %. $\left[\alpha \right]{\;}_{D}^{20}=$ +6.7° (c: 1.0, CHCl_3_). ^1^H NMR (400 MHz, DMSO-d_6_): δ 7.45 (dd, *J* = 2.0 Hz, *J* = 6.8 MHz, 1H), 7.54 (m, 10H), 6.94 (d, *J* = 8 Hz, 1H), 4.45 (d, *J* = 14, 1H), 4.16 (d, *J* = 27.2 Hz, 1H), 3.95 (s, 3H), 3.89 (s, 3H), 3.64 (d, 1H), 3.34 (d, 1H), 3.18 (d, 1H), 2.8 (m, 2H), 1.32 (s, 9H). ^13^C{1H} NMR (100 MHz, DMSO-d_6_): δ 155.2, 149.5, 146.1, 139.3, 136.1, 129.0, 128.5, 128.4, 128.1, 127.8, 127.5, 125.6, 124.3, 77.5, 71.2, 54.9, 50.9, 50.2, 35.2, 28.1. Anal. Calcd for C_30_H_38_N_2_O_7_S: C, 63.14; H, 6.71; N, 4.91; S, 5.62. Found: C, 63.17; H, 4.97; N, 4.87; S, 5.69.

**General procedure for the preparation of *n*-((2*R*,3*S*)-3-amino-2-hydroxy-4-phenylbutyl)-*n*-benzyl-R-benzenesulfonamide (6).** To a stirred solution of **5a**–**c** (0.78 mmol) in anhydrous CH_2_Cl_2_ (30 mL), trifluoroacetic acid (13 mL) was added at room temperature. After 1 hour, the reaction mixture was concentrated and treated with toluene (3 × 20 mL), evaporated in vacuum. The crude product was treated with Et_3_N and purified by chromatography on silica gel (CHCl_3_/AcOEt 9/1).

***N*-((2*R*,3*S*)-3-Amino-2-hydroxy-4-phenylbutyl)-*n*-benzyl-4-nitrobenzenesulfonamide (6a).** Compound 6a was obtained as a white solid (CH_2_Cl_2_/EtOAc 9/1), yield 41%. $\left[\alpha \right]{\;}_{D}^{20}=$ +6.4° (c: 1.0, CHCl_3_). ^1^H NMR (400 MHz, DMSO-d_6_): δ 8.34 (d, *J* = 8.6 Hz, 2H), 8,11 (bs, 2H,), 8.06 (d, *J* = 8.6 Hz, 2H), 7.26 (m, 10H), 5.67 (d, *J* = 5.6, 1H), 4.56 (d, *J* = 16.0 Hz, 1H), 4.49 (d, *J* = 16.0 Hz, 1H), 3.96 (bs, 1H), 3.38 (m, 2H), 3.16 (dd, *J* = 14.8 Hz, *J* = 8.8 Hz, 1H), 2.87 (dd, *J* = 14.4 Hz, *J* = 7.2 Hz, 1H), 2.82 (dd, *J* = 14.2 Hz, *J* = 7.6 Hz, 1H). ^13^C{1H} NMR (100 MHz, DMSO-d_6_): δ 149.6, 145.3, 136.4, 135.9, 129.3, 128.6, 128.4, 128.1, 127.6, 126.8, 124.4, 67.8, 55.2, 51.5, 49.1, 32.8. Anal. Calcd for C_23_H_25_N_3_O_5_S: C, 60.64; H, 5.53; N, 9.22; S, 7.04. Found: C, 60.68; H, 5.59; N, 9.30; S, 7.10.

***N*-((2*R*,3*S*)-3-Amino-2-hydroxy-4-phenyl-butyl)-*n*-benzyl-4-methoxybenzenesulfonamide (6b).** Compound 6b was obtained as a white solid (CH_2_Cl_2_/EtOAc 9/1), yield 35%. $\left[\alpha \right]{\;}_{D}^{20}=$ +7.5° (c: 1.0, CHCl_3_). ^1^H NMR (400 MHz, DMSO-d_6_): δ 7.75 (d, *J* = 8.4 Hz, 2H), 7.20 (m, 10H), 6.99 (d, *J =* 8.8 Hz, 2H), 4.33 (d, *J* = 14.4 Hz, 1H), 4.17 (d, *J* = 14.4Hz, 1H), 4.00 (m, 1H), 3.88 (s, 3H), 3.50 (m, 2H), 3.45 (m, 2H), 2.79 (m, 2H). ^13^C{1H} NMR (100 MHz, DMSO-d_6_): δ 136.5, 130.8, 129.3, 128.5, 128.3, 128.0, 127.3, 126.7, 114.4, 68.3, 56.0, 55.7, 51.8, 49.5, 32.5. Anal. Calcd for C_24_H_28_N_2_O_4_S: C, 65.43; H, 6.41; N, 6.36; S, 7.28. Found: C, 65.45; H, 6.46; N, 6.40; S, 7.25.

***N*-((2*R*,3*S*)-3-Amino-2-hydroxy-4-phenyl-butyl)-*n*-benzyl-3,4-methoxybenzenesulfonamide (6c).** Compound 6c was obtained as a white solid (CH_2_Cl_2_/EtOAc 9/1), yield 52%. $\left[\alpha \right]{\;}_{D}^{20}=$ +8.3° (c: 1.0, CHCl_3_). ^1^H NMR (400 MHz, DMSO-d_6_): δ 7.41 (dd, *J =* 8 Hz, *J =* 2.1 Hz, 1H), 7.15 (m, 10H), 6.9 (d, *J* =8.4 Hz, 2H), 4.35 (d, *J* = 14.8 Hz, 2H), 4.18 (d, *J* = 14.8 Hz, 2H), 3.98 (s, 1H), 3.92 (s, 3H), 3.82 (s, 3H), 3.56 (s, 1H), 3.25 (m, 2H), 2.82 (m, 2H). ^13^C{1H} NMR (100 MHz, DMSO-d_6_): δ 135.9, 130.5, 128.7, 128.2, 127.9, 127.6, 126.9, 126.5, 113.5, 67.9, 56.2, 55.8, 55.3, 51.1, 48.9, 31.8. Anal. Calcd for C_25_H_30_N_2_O_5_S: C, 63.81; H, 6.43; N, 5.95; S, 6.81. Found: C, 63.85; H, 6.41; N, 5.89; S, 6.75.

**General procedure for the preparation of *n*-((2*S*,3*R*)-3-hydroxy-4-(*n*-benzyl-arylsulfonamido)-1-phenylbutan-2-yl)heteroarene-5-carboxamide (7, 8, 9).** To a solution of 5-heterobenzoic acid (0.13 mmol), EDCI (0.20mmol), HOBt (0.20 mmol) in anhydrous CH_2_Cl_2_, a solution of amine **6a**–**c** (0.13 mmol) and diisopropylethylamine (0.78 mmol) in anhydrous CH_2_Cl_2_ was added at 0 °C under an argon atmosphere and it was stirred for 16 h at room temperature. The reaction mixture was quenched with water and extracted with CH_2_Cl_2_. The organic layers were dried on Na_2_SO_4_ and filtered and concentrated under reduced pressure. The residue was purified by silica gel column chromatography (CH_2_Cl_2_/AcOEt 9/1) to furnish inhibitors **7a**–**c**, **8a**–**c**, **9a**–**c.**

***N*-((2*S*,3*R*)-3-hydroxy-4-(*n*-benzyl-4-nitrophenylsulfonamido)-1-phenylbutan-2-yl)benzo[*b*]thiophene-5-carboxamide (7a).** Following the general procedure, compound **7a** was obtained as a white solid, yield 50%. $\left[\alpha \right]{\;}_{D}^{20}=$ +14.5° (c: 1, CHCl_3_). ^1^H NMR (400 MHz, CDCl_3_): δ 8.29 (d, *J* = 8.8 Hz, 1H), 7.95 (m, 4H), 7.54 (d, *J* = 6.4 Hz, 1H), 7.44 (d, *J* = 8.4 Hz, 1H), 7.75 (m, 12H), 6.07 (d, *J* = 8.0 Hz, 1H), 4.43 (m, 2H), 4.20 (m, 1H), 3.66 (m, 1H), 3.36 (m, 2H), 3.98 (m, 2H). ^13^C{1H} NMR (100 MHz, CDCl_3_): δ 168.6, 150.0, 144.9, 143.0, 139.4, 137.1, 135.0, 129.8, 129.3, 129.0, 128.8, 128.7, 128.4, 123.3, 126.8, 124.3, 124.1, 122.7, 122.1, 71.7, 54.9, 53.6, 51.5, 35.3. Calcd for C_32_H_29_N_3_O_6_S_2_: C, 62.42; H, 4.75; N, 6.82; S, 10.42. Found: C, 62.49; H, 4.81; N, 6.74; S, 10.35.

***N*-((2*S*,3*R*)-3-hydroxy-4-(*n*-benzyl-4-methoxyphenylsulfonamido)-1-phenylbutan-2-yl)benzo[*b*]thiophene-5-carboxamide (7b).** Following the general procedure, compound **7b** was obtained as a white solid, yield 57%. $\left[\alpha \right]{\;}_{D}^{20}=$ +1.5° (c: 0.32, CHCl_3_). ^1^H NMR (400 MHz, CDCl_3_): δ 7.98 (s, 1H), 7.88 (d, *J* = 8.4 Hz, 1H), 7.71 (d, *J* = 9.2 Hz, 2H), 7.53 (d, *J* = 5.6 Hz, 1H), 7.46 (d, *J* = 8.4 Hz, 1H), 7.37 (d, *J* = 5.2 Hz, 1H), 7.20 (m, 10H), 6.94 (d, *J* = 9.2 Hz, 2H), 6.03 (d, *J* = 8.0 Hz, 1H), 4.25 (m, 3H), 3.86 (s, 3H), 3.50 (m, 1H), 3.34 (m, 1H), 3.08 (m, 2H), 3.98 (m, 1H). ^13^C{1H} NMR (100 MHz, CDCl_3_): δ 168.1, 163.1, 142.8, 139.3, 137.5, 135.9, 130.1, 129.8, 129.4, 129.4, 128.8, 128.7, 128.5, 128.1, 127.9, 126.6, 124.1, 121.6, 122.5, 122.2, 114.4, 72.0, 55.6, 54.5, 54.2, 52.5, 35.2. Calcd for C_33_H_32_N_2_O_5_S_2_: C, 65.98; H, 5.37; N, 4.66; S, 10.68. Found: C, 65.95; H, 5.41; N, 4.62; S, 10.72.

***N*-((2*S*,3*R*)-3-hydroxy-4-(*n*-benzyl-3,4-dimethoxyphenylsulfonamido)-1-phenylbutan-2-yl)benzo[*b*]thiophene-5-carboxamide (7c).** Following the general procedure, compound **7c** was obtained as a white solid, yield 55%. $\left[\alpha \right]{\;}_{D}^{20}=$ +4.6° (c: 0.1, CHCl_3_). ^1^H NMR (400 MHz, CDCl_3_): δ 7.99 (d, *J* = 1.6 Hz, 1H), 7.87 (d, *J* = 8.8 Hz, 1H), 7.45 (m, 15H), 6.88 (d, *J =* 8.4 Hz, 1H), 6.09 (d, *J =* 8.4 Hz, 1H), 4.13 (m, 3H), 3.98 (s, 3H), 3.79 (s, 3H), 3.58 (m, 1H), 3.38 (m, 1H), 3.06 (m, 2H), 2.98 (m, 1H). ^13^C{1H} NMR (100 MHz, CDCl_3_): δ 168.1, 152.8, 149.2, 142.8, 139.3, 137.5, 135.9, 130.0, 129.4, 128.8, 128.7, 128.6, 128.1, 128.0, 126.6, 124.2, 122.6, 122.2, 121.2, 110.7, 109.6, 72.0, 56.2, 56.1, 54.4, 54.3, 52.3, 35.2. Anal. Calcd for C_34_H_34_N_2_O_6_S_2_: C, 64.74; H, 5.43; N, 4.44; S, 10.17. Found: C, 64.68; H, 5.41; N, 4.40; S, 10.11.

***N*-((2*S*,3*R*)-3-hydroxy-4-(*n*-benzyl-4-nitrophenylsulfonamido)-1-phenylbutan-2-yl)benzofuran-5-carboxamide** (**8a**). Following the general procedure, compound **8a** was obtained as a white solid, yield 53%. $\left[\alpha \right]{\;}_{D}^{20}=$ +3.1° (c: 0.22, CHCl_3_). ^1^H NMR (400 MHz, CDCl_3_): δ 8.18 (d, *J =* 8.8 Hz, 2H), 7.98 (d, *J =* 8.8 Hz, 2H), 8.80 (s, 1H), 8.70 (s, 1H), 7.45 (m, 12H), 6.82 (s, 1H), 6.03 (d, *J =* 8.0 Hz, 1H), 4.25 (m, 2H), 4.16 (m, 1H), 3.67 (m, 1H), 3.17 (m, 2H), 3.01 (m, 2H).^13^C{1H} NMR (100 MHz, CDCl_3_): δ 168.6, 156.7, 150.0, 146.5, 137.1, 135.0, 129.2, 128.8, 128.7, 128.4, 128.3, 127.6, 126.8, 124.3, 123.1, 120.7, 111.5, 71.7, 55.0, 53.5, 51.5, 35.3. Calcd for C_32_H_29_N_3_O_7_S: C, 64.09; H, 4.87; N, 7.01; S, 5.35. Found: C, 64.12; H, 4.81; N, 7.10; S, 5.39.

***N*-((2*S*,3*R*)-3-hydroxy-4-(*n*-benzyl-4-methoxyphenylsulfonamido)-1-phenylbutan-2-yl)benzofuran-5-carboxamide (8b).** Following the general procedure, compound **8b** was obtained as a white solid, yield 54%. $\left[\alpha \right]{\;}_{D}^{20}=$ +3.6° (c: 1, CHCl_3_). ^1^H NMR (400 MHz, CDCl_3_): δ 7.79 (s, 1H), 7.70 (m, 1H), 7.47 (m, 2H), 7.18 (m, 10H); 6.95 (d, *J* = 8.4 Hz, 2H), 6.81 (m, 1H), 6.00 (d, *J* = 8 Hz, 1H), 4.25 (m, 3H), 3.87 (s, 3H), 3.6 (m, 1H), 3.55 (m,1H), 3.35 (m, 1H), 3.18 (m, 2H), 2.98 (m, 1H). ^13^C{1H} NMR (100 MHz, CDCl_3_): δ 168.2, 163.1, 156.6, 146.4, 137.5, 135.9, 129.4, 129.4, 129.3, 128.8, 128.6, 128.5, 128.0, 127.5, 126.5, 123.2, 120.6, 114.6, 114.4, 111.4, 106.9, 72.6, 55.6, 54.5, 54.2, 52.5, 35.2, 31.9. Calcd for C_33_H_32_N_2_O_6_S: C, 67.79; H, 5.52; N, 4.79; S, 5.48. Found: C, 67.75; H, 5.60; N, 4.73; S, 5.54.

***N*-((2*S*,3*R*)-3-hydroxy-4-(*n*-benzyl-3,4-dimethoxyphenylsulfonamido)-1-phenylbutan-2-yl)benzofuran-5-carboxamide (8c).** Following the general procedure, compound **8c** was obtained as a white solid, yield 56%. $\left[\alpha \right]{\;}_{D}^{20}=$ +19.5° (c: 1, CHCl_3_). ^1^H NMR (400 MHz, CDCl_3_): δ 7.80 (s, 1H), 7.6 (d, *J* = 2.0 Hz, 1H), 7.43 (m, 3H), 7.20 (m, 12H), 6.89 (d, *J* = 8.4 Hz, 1H), 6.8 (d, *J* = 2.1 Hz, 1H), 6.1 (d, *J* = 7.6Hz, 1H), 3.93 (s, 3H), 3.80 (s, 3H), 3.52 (m, 1H), 3.37 (m, 1H), 3.06 (m, 2H), 2.92 (m, 1H). ^13^C{1H} NMR (100 MHz, CDCl_3_): δ 168.2, 156.6, 152.8, 130.1, 129.4, 128.9, 128.8, 128.7, 128.5, 128.1, 127.5, 126.6, 123.2, 121.1, 120.7, 111.6, 110.7, 109.6, 106.9, 72.0, 56.2, 56.1, 54.3, 52.4, 35.2, 30.9. Calcd for C_34_H_34_N_2_O_7_S: C, 66.43; H, 5.57; N, 4.56; S, 5.22. Found: C, 66.45; H, 5.61; N, 4.50; S, 5.25.

***N*-((2*S*,3*R*)-3-hydroxy-4-(*n*-benzyl-4-nitrophenylsulfonamido)-1-phenylbutan-2-yl)1H-indol-5-carboxamide (9a).** Following the general procedure, compound **9a** was obtained as a white solid, yield 33%. $\left[\alpha \right]{\;}_{D}^{20}=$ +25.8° (c: 1.3, CHCl_3_). ^1^H NMR (400 MHz, CDCl_3_): δ 8.64 (s, 1H), 7.84 (s, 1H), 7.72 (d, *J* = 8.8 Hz, 2H), 7.40-7.15 (m, 14H), 6.93 (d, *J* = 8.8 Hz, 2H), 6.59 (s, 1H), 6.03 (d, *J* =6 Hz, 1H), 4.25 (m, 2H), 3.84 (s, 3H), 3.50 (m, 1H), 3.35 (dd, *J* = 15 Hz, *J* = 4.4 Hz, 1H), 3.17 (m, 2H), 2.90 (m, 2H). ^13^C{1H} NMR (100 MHz, CDCl_3_): δ 169.2, 163.0, 137.7, 136.6, 136.0, 129.5, 129.4, 128.8, 128.7, 128.5, 128.0, 127.4, 126.5, 125.7, 120.8, 120.4, 114.4, 111.0, 103.6, 72.1, 55.6, 54.5, 54.2, 52.5, 35.3. Calcd for C_32_H_30_N_4_O_6_S: C, 64.20; H, 5.05; N, 9.36; S, 5.36. Found: C, 64.25; H, 4.81; N, 9.30; S, 5.39.

***N*-((2*S*,3*R*)-3-hydroxy-4-(*n*-benzyl-4-methoxyphenylsulfonamido)-1-phenylbutan-2-yl)1H-indol-5-carboxamide (9b).** Following the general procedure, compound **9b** was obtained as a white solid, yield 43%. $\left[\alpha \right]{\;}_{D}^{20}=$ +10.2° (c: 0.4, CHCl_3_). ^1^H NMR (400 MHz, CDCl_3_): δ 8.47 (s, 1H) 8.24 (d, *J* = 8.8 Hz, 2H), 7.90(d, *J* = 8.8 Hz, 2H), 7.81 (s, 1H), 7.37-7.17 (m, 14H), 6.59 (s, 1H), 6.04 (d, *J* = 7.2 Hz, 1H), 4.52 (d, *J* = 14.4 Hz, 1H), 4.37 (d, *J* =14.4 Hz, 1H), 4.18 (m, 2H), 3.81-3.24 (m, 2H), 2.95 (m, 2H). ^13^C{1H} NMR (100 MHz, CDCl_3_): 169.7, 149.9, 145.0, 137.7, 137.2, 135.1, 129.3, 128.8, 128.7, 128.6, 128.4, 128.3, 127.5, 126.8, 125.8, 125.3, 124.3, 120.9, 120.4, 111.1, 103.7, 71.8, 55.1, 53.4, 51.3, 35.6. Calcd for C_33_H_33_N_3_O_5_S: C, 67.90; H, 5.70; N, 7.20; S, 5.49. Found: C, 67.96; H, 5.76; N, 7.22; S, 5.56.

***N*-((2*S*,3*R*)-3-hydroxy-4-(*n*-benzyl-3,4-dimethoxyphenylsulfonamido)-1-phenylbutan-2-yl)1H-indol-5-carboxamide (9c).** Following the general procedure, compound **9c** was obtained as a white solid, yield 44%. $\left[\alpha \right]{\;}_{D}^{20}=$ +18.7° (c: 1, CHCl_3_). ^1^H NMR (400 MHz, CDCl_3_): δ 8.76 (bs, 1H), 7.85 (s, 1H), 7.37 (m, 3H), 7.23 (m, 11H), 6.85 (d, *J* = 8.4Hz, 1H), 6.56 (s, 1H), 6.12 (d, *J* = 8Hz, 1H), 4.37 (d, *J* = 14.8Hz, 1H), 3.90 (s, 3H), 3.76 (s, 3H), 3.55 (m, 1H), 3.40 (dd, *J* = 12Hz, *J* = 4Hz, 1H), 3.10 (m, 2H), 2.92 (m, 1H). ^13^C{1H} NMR (100 MHz, CDCl_3_): δ 169.2, 152.6, 149.1, 137.6, 135.8, 130.1, 129.3, 128.7, 128.6, 128.4, 127.9, 127.7, 127.4, 126.4, 125.7, 125.3, 121.1, 120.7, 120.2, 110.9, 110.6, 109.6, 103.4, 72.0, 60.3, 56.1, 55.9, 54.4, 54.1, 52.3, 35.2, 31.4, 29.5, 22.5, 20.9, 14.0. Calcd for C_34_H_35_N_3_O_6_S: C, 66.54; H, 5.75; N, 6.85; S, 5.22. Found: C, 66.62; H, 5.81; N, 6.80; S, 5.27.

**(2*R*,3*S*)-3-amino-1-(benzylamino)-4-phenylbutan-2-ol (10).** A solution of (2***S***,3***S***)-1,2-epoxy-3-(Boc-amino)-4-phenylbutane **3** (1.6 mmol) and benzylamine (1.5 mmol) in *i*-PrOH (10 mL) was stirred under reflux for 16 h. The reaction mixture was rotary evaporated, and the crude product was purified by recrystallization in methanol/water (7:3) to afford compound **4** as a white solid. Then product **4** (1 mmol) was dissolved in MeCN (10 ml) and tosic acid monohydrate was added (3 mmol); the resulting mixture was stirred at room temperature for 5 h. The precipitate formed was filtered off and washed with Et_2_O to give compound **10** as a white solid, 60% yield. ^1^H NMR (400 MHz, DMSO-d_6_): ***δ*** 9.00 (bs, 1H), 8.89 (bs, 1H), 7.97 (bs, 3H), 7.49 (d, *J* = 7.6 Hz, 4H), 7.45 (m, 5H), 7.30 (m, 5H), 7.13 (d, *J* = 7.6 Hz, 4H), 6.11 (bs, 1H), 4.15 (m, 2H), 4.06 (d, *J* = 10.4 Hz, 1H), 3.53 (m, 1H), 3.13 (m, 1H), 2.86 (m, 3H), 2.29 (s, 6H). ^13^C{1H} NMR (100 MHz, DMSO-d_6_): δ 145.3, 137.9, 135.9, 131.3, 130.2, 129.3, 129.1, 128.9, 128.7, 128.1, 127.0, 125.5, 65.7, 54.9, 50.2, 47.3, 33.1, 20.8. Calcd for C_17_H_22_N_2_O: C, 75.52; H, 8.20; N, 10.36. Found: C, 75.59; H, 8.28; N, 10.28. 

### 2.2. In Vitro Activity Test

IC50 values were determined at pH 5.5 using recombinant wild-type HIV-1 PR from Bachem and the fluorogenic substrate Abz-Thr-Ile-Nle-Phe(p-NO2)-Gln-Arg-NH2 (Abz-NF⁄-6; Bachem AG, Bubendorf, CH). Darunavir was used in this assay as a reference inhibitor for titration of the active enzyme. The assay was performed as follows: **Dilution Buffer:** 100 mM MES buffer, pH 5.5, containing 400 mM NaCl, 1 mM EDTA, 1 mM DTT and 1 mg/mL BSA. **Solution A:** 10 μL of a substrate stock solution in DMSO (10 mg/ml, 10.6 mM) were diluted in 1.99 ml of dilution buffer to a final concentration of 53mM. **Solution B:** 10 μL of a protease stock solution (0.4 mg/mL) in 10 mM sodium phosphate buffer, pH 6.5, containing 1 mM EDTA, 10% glycerol, 0.05% mercaptoethanol, 50 mM NaCl, were diluted 100 times with the dilution buffer, pH 5.5, to a final concentration of 0.004 mg/ml. 

**Assay**: 114 μL of solution A, 11 μL of solution B and 75 μL of the dilution buffer were pre-incubated in a cuvette at 25 °C, and the fluorescence was recorded at 325 nm excitation and 420 nm emission for 10 min. A total of 2 μL of the inhibitor in DMSO was then added, and the fluorescence was recorded for a further 10 min. Final concentrations in the assay were 1.2 nM protease, 30 μM substrate and 0.1–10 μM inhibitor. IC50 was obtained by measuring the relative residual enzyme activity (ratio of the increase of fluorescence velocities before and after the addition of inhibitor) and by fitting the residual activity vs. inhibitor concentration semilog plots to a tetraparametric logistic function (Sigma plot 2001, SPSS Inc., Chicago, IL, USA). All the measures were triplicated.

### 2.3. Molecular Modeling

A crystallographic structure of the wt-HIV-Pr complex with darunavir (PDB id. 4LL3) was used as the starting geometry of the model complexes. The structure was prepared by adding hydrogen atoms, removing water crystallization molecules but keeping the essential one inside the catalytic site and always choosing the most symmetrical option for amino acid side chains, allowing more solutions. The structure was then optimized with the Amber* force field as implemented in the Schrödinger suite [26,27]. After docking, the complexes were thermalized by a MD run carried out with Yasara (NTV, 300 °K, 500 ps) and finally optimized as previously described. The models of the heterocycle–methane complexes were obtained at the MO62X/6-311++G(d,p) level with Gaussian 09 [28].

## 3. Results and Discussion

### 3.1. Chemistry

**Scheme 1:**
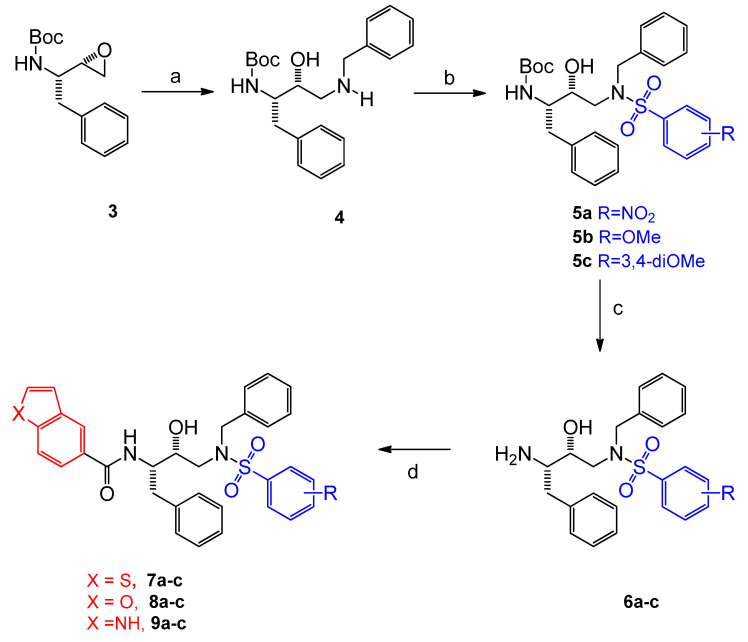
Synthesis of inhibitors **7a**–**c**, **8a**–**c** and **9a**–**c**: (a) BnNH_2_, *i*-PrOH, 60°C, 4h (88% yield); (b) arylsulfonyl chloride, Et_3_N, CH_2_Cl_2_, rt, 24h (5a 94%; 5b 85%; 5c 90%); (c) TFA/CH_2_Cl_2_ 30%, rt, 1 h; then Et_3_N; (d) 5-heteroarylcarboxylic acid, EDC, HOBt, then 6a–c, *i*Pr_2_NEt, CH_2_Cl_2_, 24h, rt (**7a**, 50%; **7b** 57%; **7c** 55%; **8a** 53%; **8b** 54%; **8c** 56%; **9a** 33%; **9b** 43%; **9c** 44%).

The preparation of aromatic sulfonamides (general structure **A**, Figure 2) started from homochiral *N*-Boc-protected amino epoxide **3**, keeping the established stereochemistry during the synthesis [29,30]. The epoxide was firstly opened with benzylamine to afford the monoprotected diaminoalcohol **4**. Then, the substituted benzenesulfonyl groups were introduced, and the *N*-Boc group was efficiently displaced by treatment with trifluoroacetic acid in dichloromethane. The crude ammonium trifluoroacetate derivatives were treated with NEt_3_, affording the free amines **6a**–**c**. The amines were reacted with 5-heteroarylcarboxylic acids, previously activated with 1-ethyl-3-(3-dimethylaminopropyl)carbodiimide and hydroxybenzotriazole. Thus, the final products **7a**–**c, 8a**–**c** and **9a**–**c** were obtained in four steps and with excellent overall yield (Scheme 1).

This synthetic pathway appears very solid, high-yielding and general, irrespective of the *N*-group, the sulfonamide or the type of heteroaryl moiety chosen. The easy access of substrates represents an open door to molecules with synergic biological activity, as anticancer activity, especially because there has been growing interest in repurposing PIs for the treatment of cancer [31]. 

**Scheme 2:**
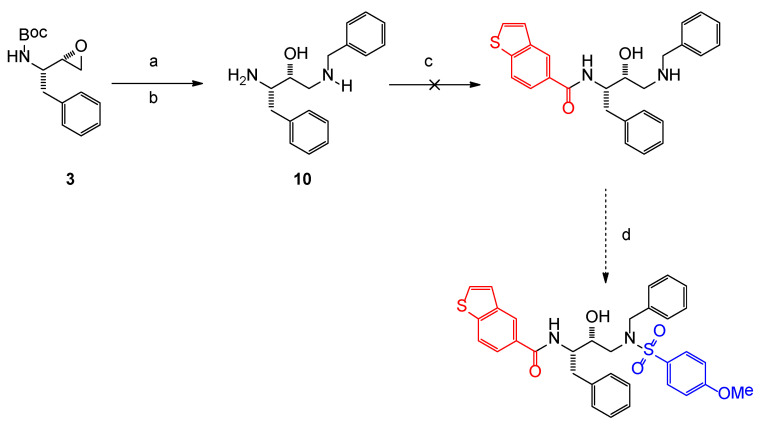
(a) BnNH_2_, *i*-PrOH, 60 °C, 4 h; (b) ***p***-TsOH, MeCN, 5 h, rt (60% yield from **3**); (c) 5-heteroarylcarboxylic acid, EDC, HOBt, then **10**, *i*Pr_2_NEt, CH_2_Cl_2_, 24 h, rt; (d) p-methoxybenzene sulfonyl chloride, Et_3_N, CH_2_Cl_2_.

Despite the fact that this pathway proved to be solid, diversity-oriented synthesis was studied to introduce different functionalities according to needs. In particular, the removal of the Boc group immediately after opening the commercial epoxide **3** with benzylamine allowed to diaminoalcohol **10**. In this way, it should be possible to firstly introduce the desired heteroaryl moiety on the primary amine and then the different aromatic sulfonyls on the sterically hindering secondary amine. Unfortunately, this strategy proved to not be applicable because, under these conditions, diamine 10 did not react to afford the desired heterocarboxyamide derivative (Scheme 2). 

### 3.2. In Vitro Activity 

IC_50_ values were obtained on recombinant wild-type HIV protease by measuring the initial rates of hydrolysis of the fluorogenic substrate Abz-Thr-Ile-Nle-Phe(NO_2_)-Gln-Arg (Table 1) [13,14,15,16,17,18]. The initial rates of the enzyme-catalyzed reactions were measured at different inhibitors concentrations, while the reference rates for the not-inhibited reactions were measured each time before the addition of the inhibitors. IC50 was obtained by measuring the relative residual enzyme activity (ratio of the increase of fluorescence velocities before and after the addition of inhibitor) and by fitting the residual activity vs. inhibitor concentration semilog plots to a tetraparametric logistic function. The results are the mean of three independent experiments and are reported in Table 1, while the plots are reported in Supplementary Figure S1.

All the inhibitors proved to be active and capable of reducing the enzyme activity to less than 10% of that of the free enzyme within the tested range of concentrations (0.5–10 μM) with similar efficacy. Their power is clearly related to the nature of the heterocyclic system at P2. The indole derivatives **9a**–**c** are the most powerful inhibitors and perform better than darunavir under our experimental conditions. When tested at 0.5 nM concentrations, they are able to inhibit most of the enzyme activity. We do not report the IC50s for the indole compounds as the residual activities measured at 0.5 nM are already less than 50%, and we are unable to measure the IC50 as the nominal concentration of the enzyme in the test is 1.2 nM, as estimated from the amount of enzyme declared by the producer in the sample. Even if this concentration is most likely overestimated, we prefer not to evaluate the IC50 for such compounds, as the values could be physically meaningless. One order of magnitude in affinity is lost on changing the indole to benzothiophene, and compounds **7a**–**c** show nanomolar IC50s. A further decrease is observed with benzofuran at P2, as in inhibitors **8a**–**c**, with tenth nanomolar IC50s. Most of the IC50s of compounds **7** and **8** are in the same order of magnitude as that of darunavir, although statistically distinguishable from it (see the *t*-test plot in Supplementary Figure S2.

A minor effect is given by the substituents at the arylsulfonamide group at P2’, where, at least in series **7** and **8**, the 4-methoxyphenyl moiety seems slightly better than 4-nitrophenyl and 3,4-dimethoxyphenyl. This effect, if present, cannot be evaluated in the more active compounds, **9a**–**c**.

The beneficial effect of indole in comparison with benzothiophene and benzofuran was already observed in our previous studies on compounds **1** and **2**, which are different at P1’ (an isobutyl group is present), and in **2** also as to the length of the chain connecting P2 with the core (a one-atom longer carbamate linker). 

### 3.3. Molecular Modeling

The models were, therefore, built to gain insight into the structural effects at the origin of the observed activities.

The optimized complexes of HIV-Pr with all the indole derivatives **9a**–**c**, **1c** and **2c** were obtained and compared with the experimental crystallographic structure of the complex of darunavir with the enzyme. The model complexes of the benzothiophene and benzofuran derivatives were then also obtained.

**Figure 3:**
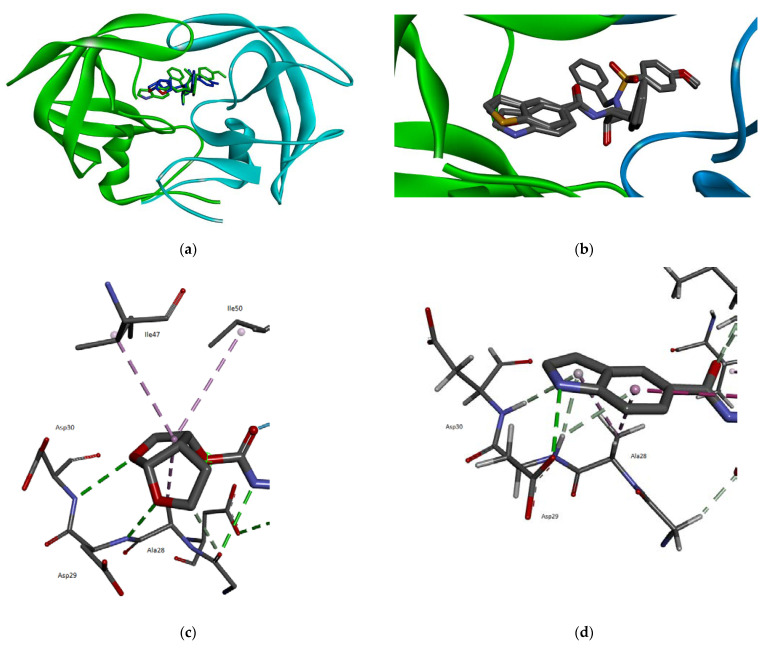
(**a**): Overlay of the crystallographic structure of the Hiv-pr complex with darunavir and the model structure of the complex of **9b**; (**b**): overlay of the model structures of the complexes of **7b**, **8b** and **9b** with the protease; (**c**): interactions of the dioxabicyclo octane group of darunavir with the protein; (**d**): interactions of the indole system of **9b** with the protein.

All the heterocyclic systems are hosted by the S2 site of the protein in a very similar way. An overlay of the structures of darunavir and **9b** is reported in Figure 3a, while the overlay of the structures of **7b**, **8b** and **9b** is reported in Figure 3b.

The heteroatoms (S, O, N) are closely superimposed, while the heterocyclic systems are quite more exposed to the solvent than the dioxabicyclo octane side chain of darunavir. Nevertheless, the indole derivatives inhibit the enzyme better than darunavir. Details of the interactions established by darunavir and **9b** are reported in Figure 3c,d.

A clear difference is given by the ability of the indole NH group to act as a hydrogen bond donor towards the carboxylate group of Asp30. This interaction cannot be established by darunavir nor by our benzofuran–benzothiophene compounds, which can only accept hydrogen bonds. However, the heteroatoms in compounds **7**, **8** and **9** point outside the binding site and are largely exposed to the solvent. Thus, the interaction with Asp30 is expected to be rather weak, and other effects are most likely operating. The aromatic rings of our inhibitors can clearly establish significant interactions mediated by their π systems. A recent study has compared, at different levels of theory, the ability of indole, thiophene and benzofuran as partners in the formation of π–π stacking interactions with DNA bases [32]. Very interestingly, indole was capable of establishing the strongest π–π stacking interactions, followed by benzothiophene and then by benzofuran. This order resembles that of the inhibitory activity of our compounds. By the way, aromatic side chains are not present in subsite S2 of HIV-Pr, rather there is a number of methyl groups wallpapering the surface of S2, and those from Ala28 and Ile47 (to a minor extent) are found to interact with the heterocyclic side chains of our compounds. We have, therefore, carried out a preliminary evaluation on the ability of indole, thiophene and benzofuran in CH_3_/π interactions by modeling their complexes with methane. We have followed one of the approaches reported by Toupkanloo and Rahmani, optimizing the structures at the MO62X/6-311++G(d,p) level, and we have actually found that the strength of the CH_3_/π interaction follows the same order found for π–π stacking. The superior performance of indole over benzofuran and thiophene is thus probably due to this effect, which is likely very general when comparing the interactions of such compounds with biomolecules. Moreover, the indole system is also capable of acting as an acceptor in π-acceptor hydrogen bonding, and we find a couple of interactions where the donors are the backbone NH of Asp29 and Asp30. These interactions replace the hydrogen bonding ones given by the backbone NH of Ala28 and Asp29 towards the oxygens in darunavir (Figure 3a,b). 

A further point in favor of **9b** in comparison with darunavir may be given by the benzyl side chain that replaces the alkyl chain of the drug at P1’. The aromatic side chain seems able to actually establish more favorable hydrophobic interactions (see the Supplementary Figures S4 and S5 with the maps of the recognized interactions). This may also explain the better performance of the set of inhibitors reported in the present paper in comparison with other sets previously described by us (namely, the difference between **9c** and **1c**). 

As to the minor effect given by the substituents at P2’, a very simple explanation is found in the relatively small size of the S2’ subsite, which can fit the aromatic ring with one methoxy group well, but is unable to host both the 3,4-dimethoxyphenly and the 4-nitrophenyl groups without suffering from conformational distortions of the ligands (Supplementary Figure S3). 

## 4. Conclusions

In conclusion, all the newly synthesized molecules with pseudo-symmetric hydroxyethylamine core*s* proved to be active, with excellent IC_50_ values and with several interactions with the enzyme site. Thus, we can highlight that the presence of a bis benzyl in the core can give rigidity to the molecules and maximize the interaction. Furthermore, the indole ring is apparently the heterocycle, which confers greater inhibitory activity; this makes compounds **9a**–**c** very promising molecules, regardless of the nature of the substituent present on the sulfonamide.

## Figures and Tables

**Table 1:** In vitro inhibition activity of compounds **7**.

Entry	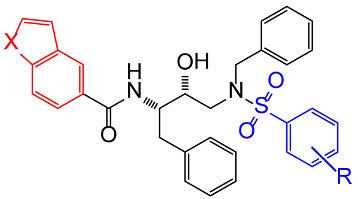 Compound	X	R	IC_50_ (nM)	Std. Error (nM)
**1**	**7a**	S	4-NO_2_	4.2	0.6
**2**	**7b**	S	4-OMe	2.3	0.4
**3**	**7c**	S	3,4-diOMe	6.2	2.1
**4**	**8a**	O	4-NO_2_	47.0	6.0
**5**	**8b**	O	4-OMe	9.6	0.1
**6**	**8c**	O	3,4-diOMe	82.0	7.0
**7**	**9a**	NH	4-NO_2_	27.7% *	3.5%
**8**	**9b**	NH	4-OMe	36.3% *	5.1%
**9**	**9c**	NH	3,4-diOMe	10.2% *	2.4%
**10**	darunavir			1.8	0.3

* residual activity at the 0.5 nM inhibitor.

## Supplementary Materials

The following are available online at https://www.mdpi.com/article/10.3390/biom11111584/s1. Copy of ^1^H and ^13^C NMR spectra of compounds **5c**, **6c, 7a–c, 8a–c** and **9a–c** inhibition assays and model structures of complex of compound **9b** and darunavir with the protease are available.
