# Supplementary material for: The Pseudo-Symmetric N-benzyl Hydroxyethylamine Core in a New Series of Heteroarylcarboxyamide HIV-1 Pr Inhibitors: Synthesis, Molecular Modeling and Biological Evaluation
Source: Biomolecules. 2021 Oct 26;11(11):1584. doi: 10.3390/biom11111584 (PMC8615997; doi:10.3390/biom11111584)
Supplement: Supplementary file 1 [file biomolecules-11-01584-s001.zip › biomolecules-1408886-SI.pdf]

Supplementary material

The pseudo-symmetric *N*-benzyl hydroxyethylamine *core* in a new series of heteroarylcarboxyamide HIV-1 Pr inhibitors: synthesis, molecular modeling and biological evaluation.

Table of contents

Copies of ^{1}H and ^{13}C NMR spectra of compounds **5c** and **6c**	**S2-S3**
Copies of ^{1}H and ^{13}C NMR spectra of compounds **7a-c, 8a-c, 9a-c**	**S4-S12**
Supplementary figures S1-S4	**S13-S17**

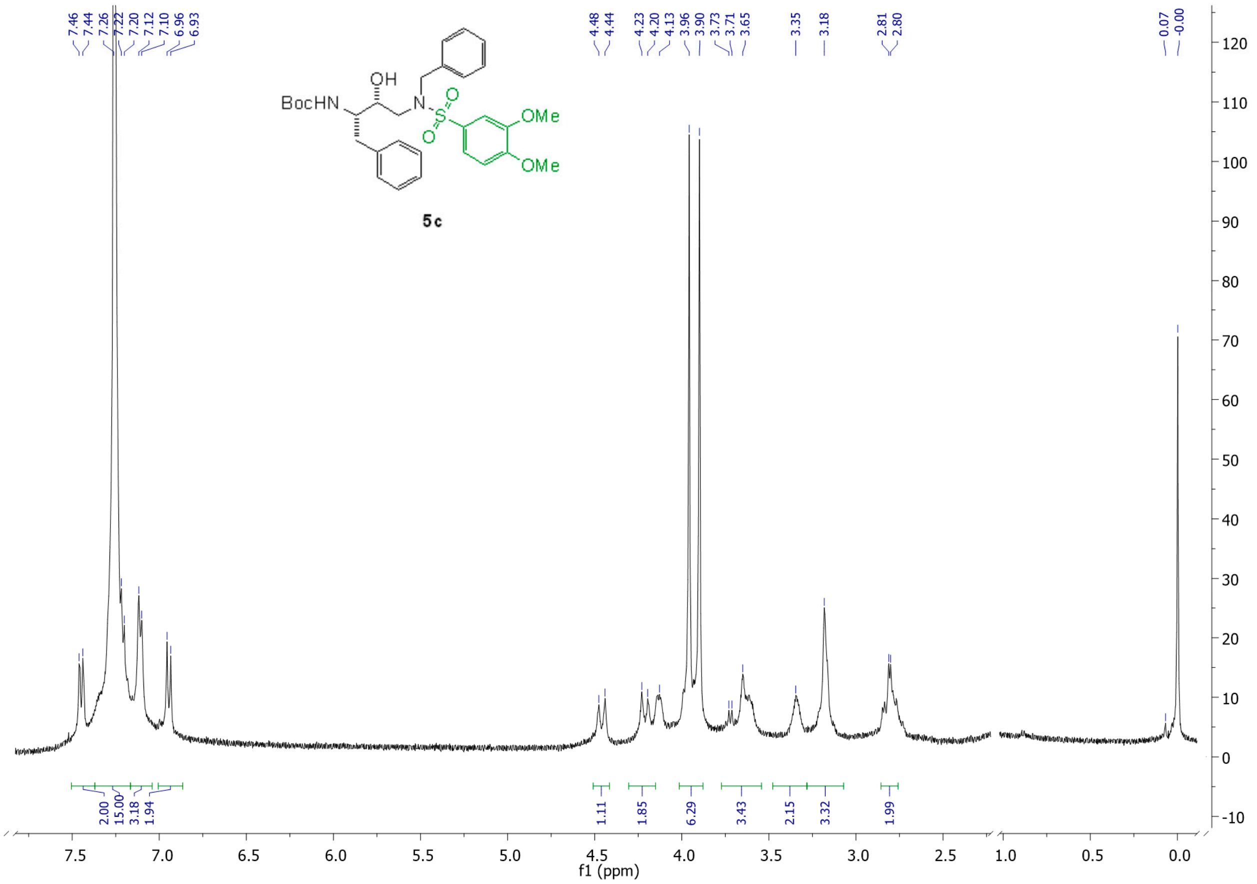
7.46 7.44 7.26 7.22 7.20 7.12 7.10 6.96 6.93
4.48 4.44 4.23 4.20 4.13 3.96 3.90 3.73 3.71 3.65 3.35 3.18 2.81 2.80
0.07 -0.00
BocHN
OH
N
S
O
O
OMe
OMe
5c
2.00 15.00 3.18 1.94 1.11 1.85 6.29 3.43 2.15 3.32 1.99
f1 (ppm)

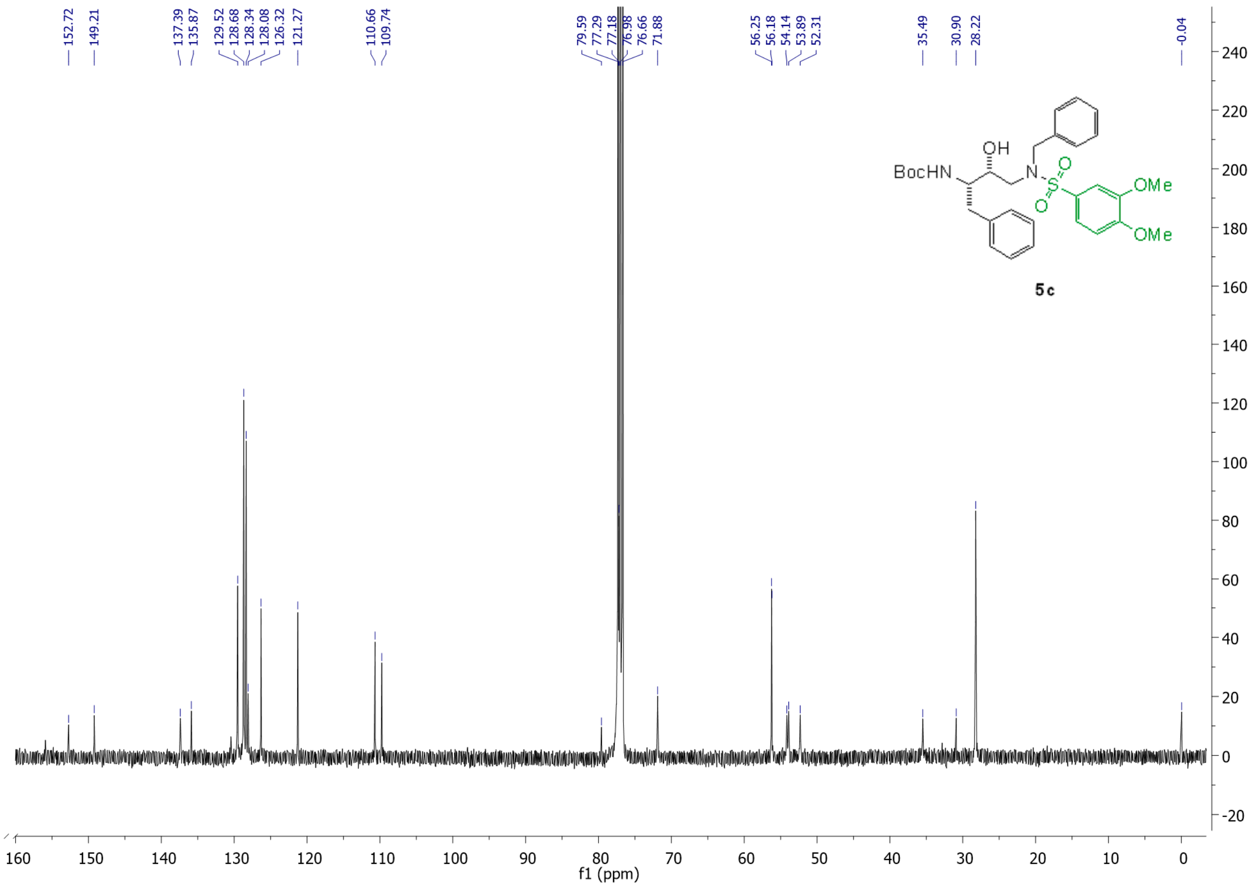
152.72 149.21 137.39 135.87 129.52 128.68 128.34 128.08 126.32 121.27 110.66 109.74
79.59 77.29 77.18 76.98 76.66 71.88 56.25 56.18 54.14 53.89 52.31 35.49 30.90 28.22 -0.04
BocHN
OH
N
S
O
O
OMe
OMe
5c
f1 (ppm)

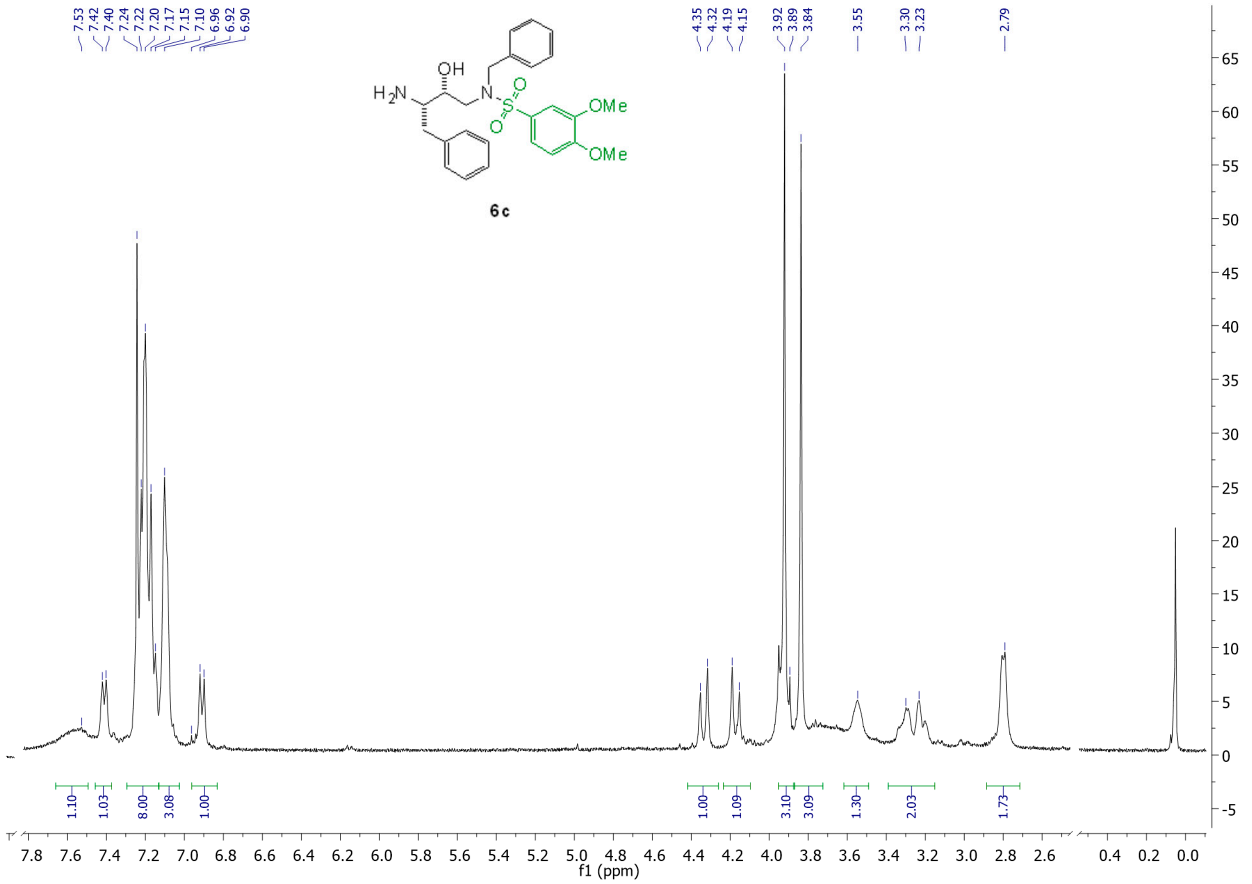
7.53 7.42 7.40 7.24 7.22 7.20 7.17 7.15 7.10 6.96 6.92 6.90
4.35 4.32 4.19 4.15 3.92 3.89 3.84 3.55 3.30 3.23 2.79
OH
H2N
N
S
O
O
OMe
OMe
6c
1.10 1.03 8.00 3.08 1.00 1.00 1.09 3.10 3.09 1.30 2.03 1.73
f1 (ppm)

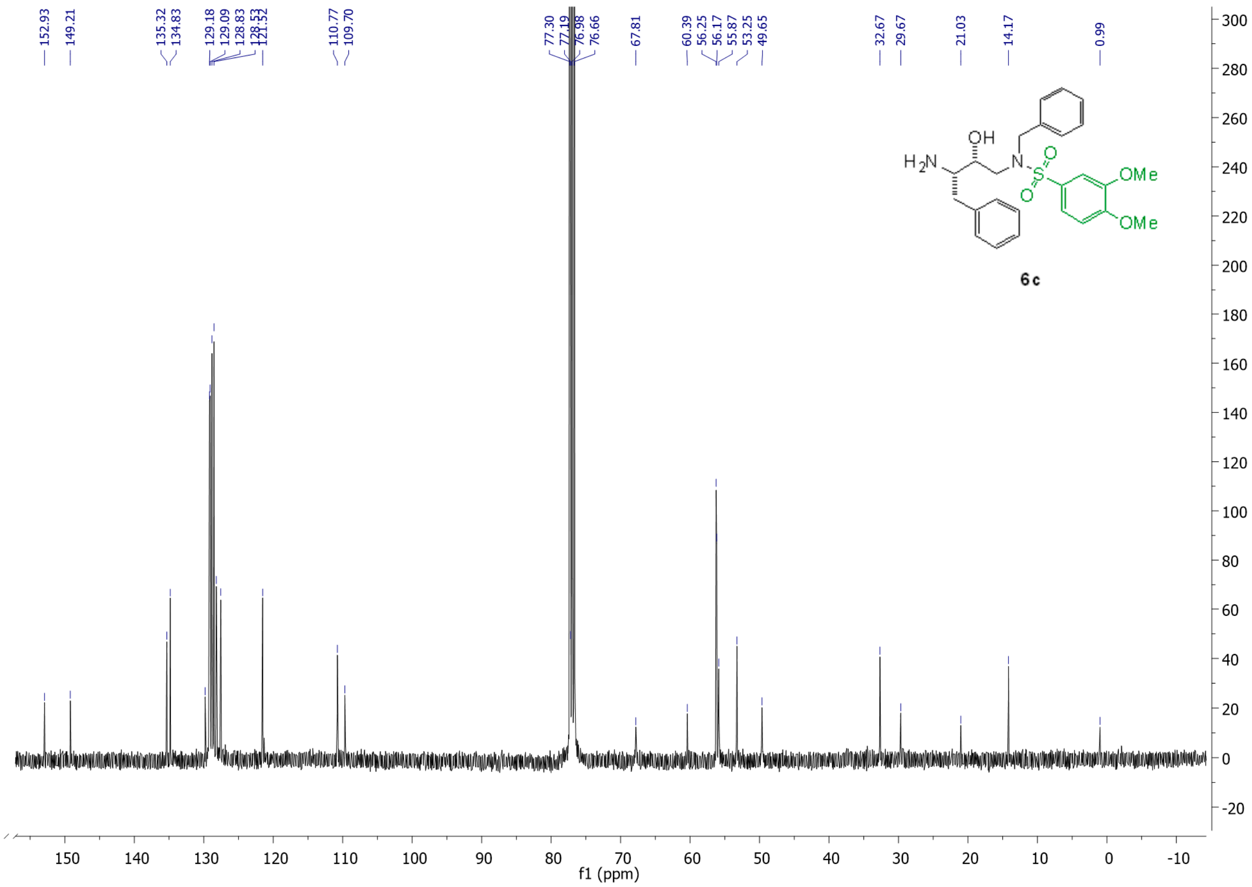
152.93 149.21 135.32 134.83 129.18 129.09 128.83 128.53 121.52 110.77 109.70 77.30 77.19 76.98 76.66 67.81 60.39 56.25 56.17 55.87 53.25 49.65 32.67 29.67 21.03 14.17 0.99
OH
H2N
N
S
O
O
OMe
OMe
6c
f1 (ppm)

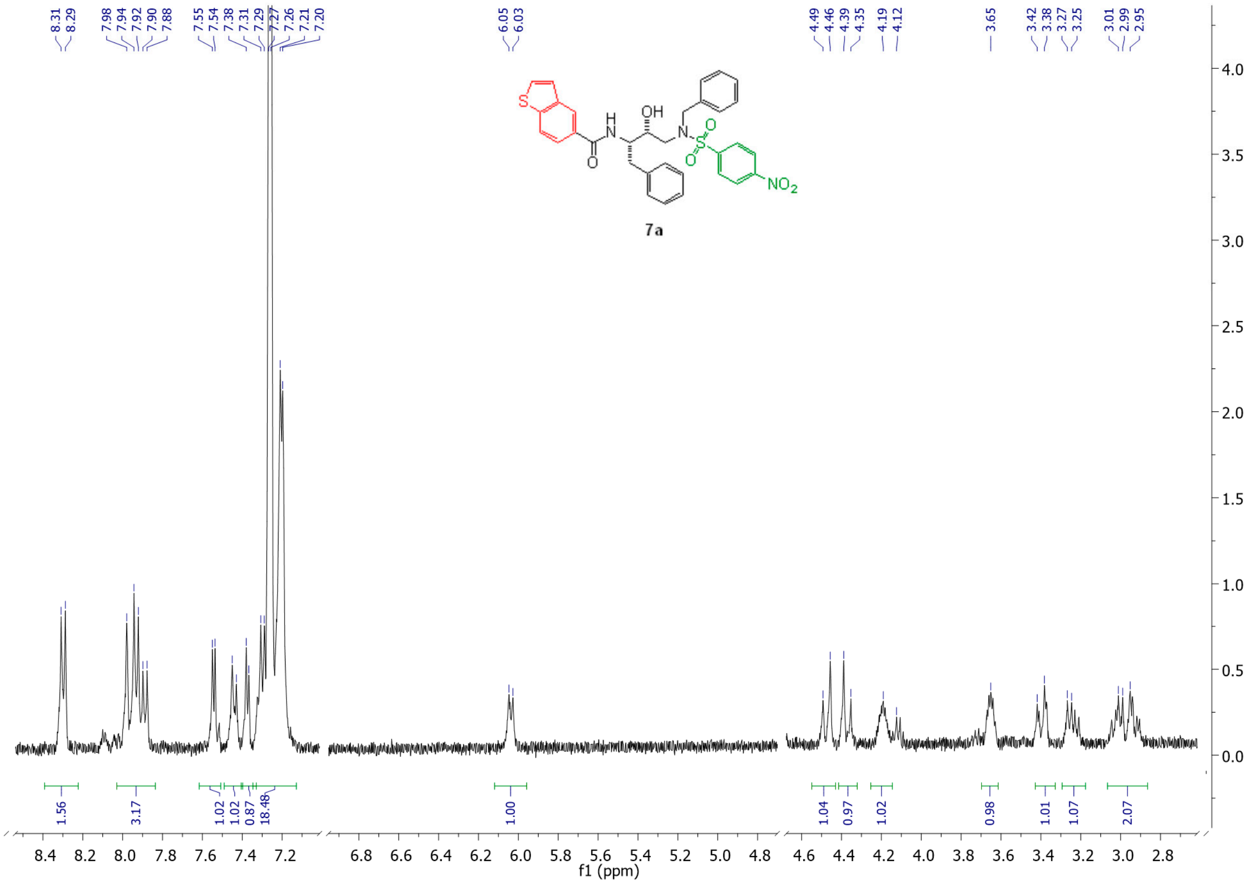
8.31
8.29
7.98
7.94
7.92
7.90
7.88
7.55
7.54
7.38
7.31
7.29
7.27
7.26
7.21
7.20
6.05
6.03
4.49
4.46
4.39
4.35
4.19
4.12
3.65
3.42
3.38
3.27
3.25
3.01
2.99
2.95
1.56
3.17
1.02
1.02
0.87
18.48
1.00
1.04
0.97
1.02
0.98
1.01
1.07
2.07
7a
f1 (ppm)

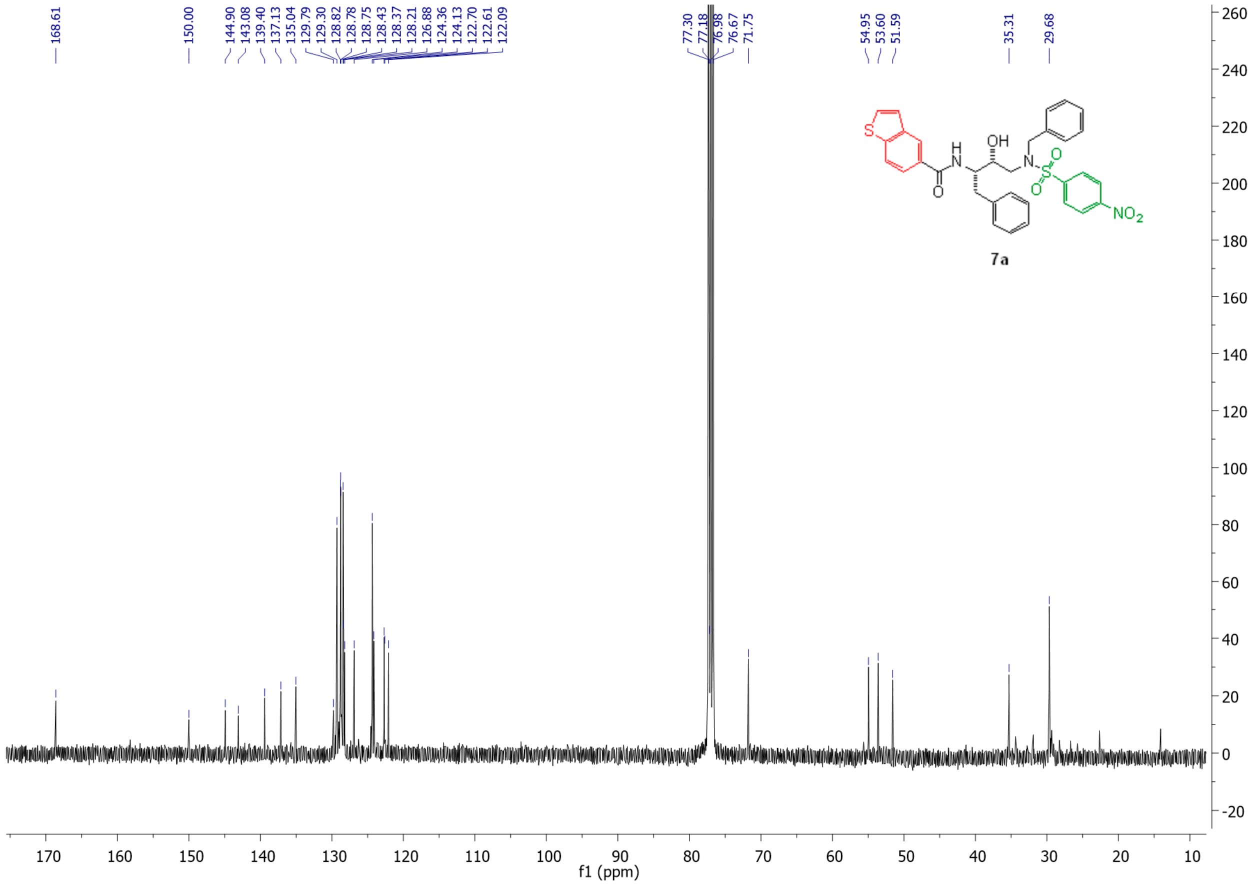
168.61
150.00
144.90
143.08
139.40
137.13
135.04
129.79
129.30
128.82
128.78
128.75
128.43
128.37
128.21
126.88
124.36
124.13
122.70
122.61
122.09
77.30
77.18
76.98
76.67
71.75
54.95
53.60
51.59
35.31
29.68
7a
f1 (ppm)

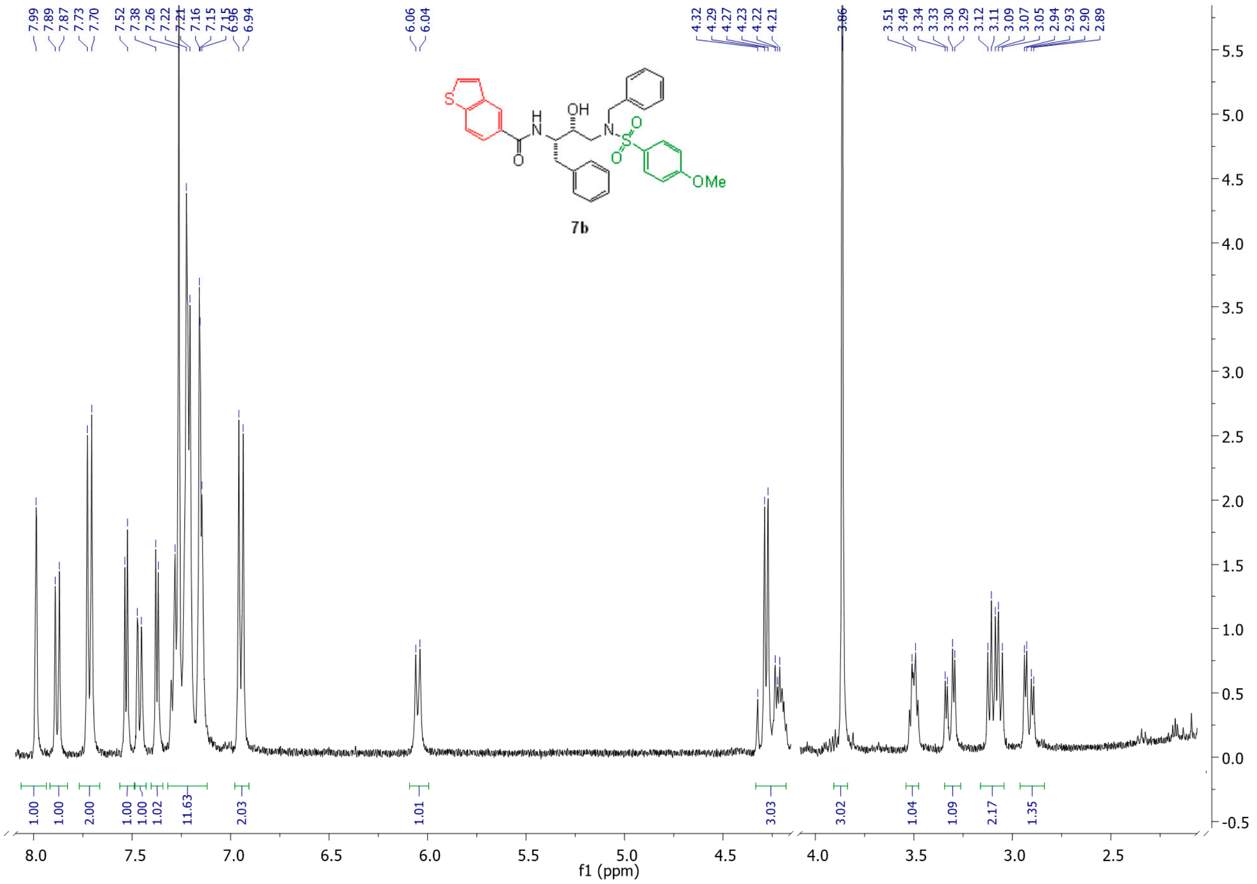
7.99
7.89
7.87
7.73
7.70
7.52
7.38
7.26
7.22
7.21
7.16
7.15
6.94
6.06
6.04
4.32
4.29
4.27
4.23
4.22
4.21
3.86
3.51
3.49
3.34
3.33
3.30
3.29
3.12
3.11
3.09
3.07
3.05
2.94
2.93
2.90
2.89
OH
H
N
S
O
OMe
7b
f1 (ppm)

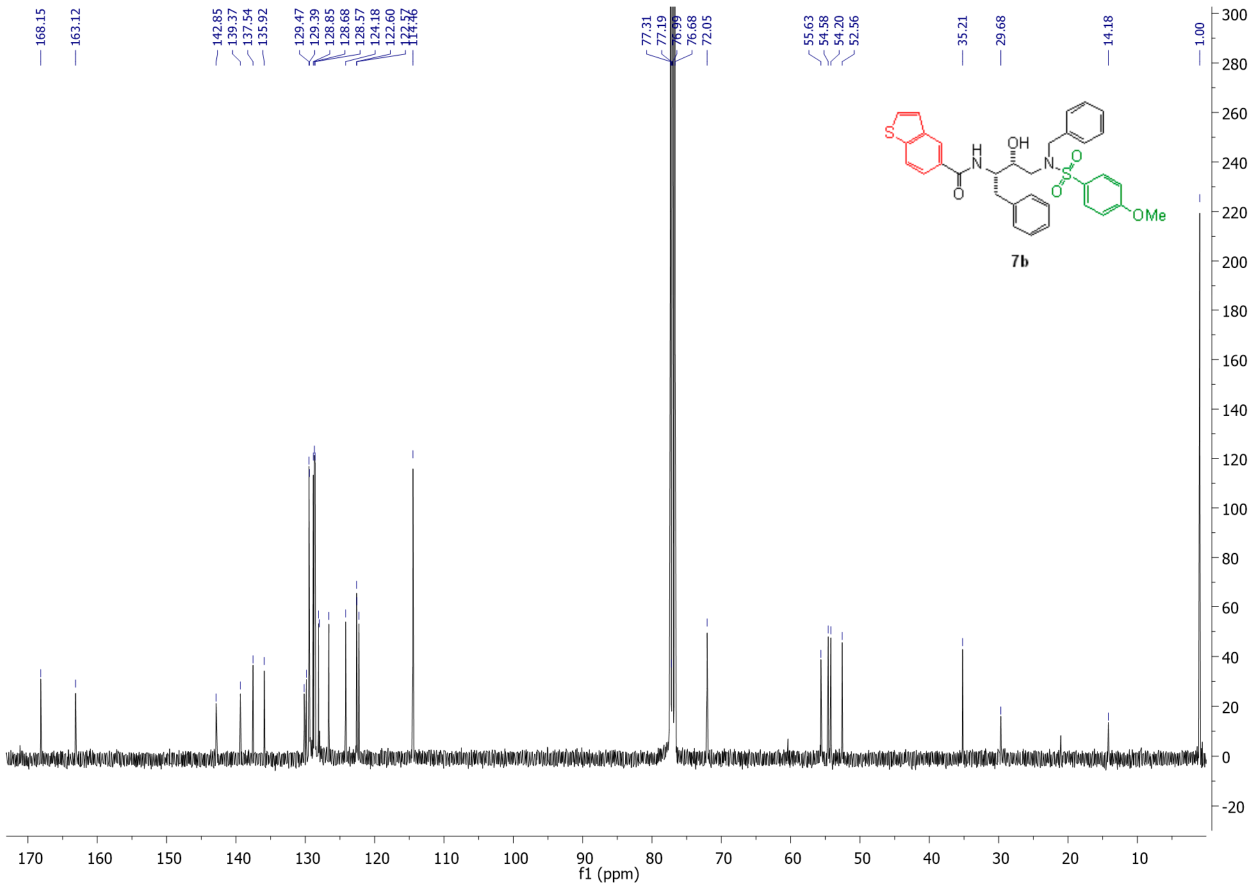
168.15
163.12
142.85
139.37
137.54
135.92
129.47
129.39
128.85
128.68
128.57
124.18
122.60
114.46
77.31
77.19
76.68
72.05
55.63
54.58
54.20
52.56
35.21
29.68
14.18
1.00
OH
H
N
S
O
OMe
7b
f1 (ppm)

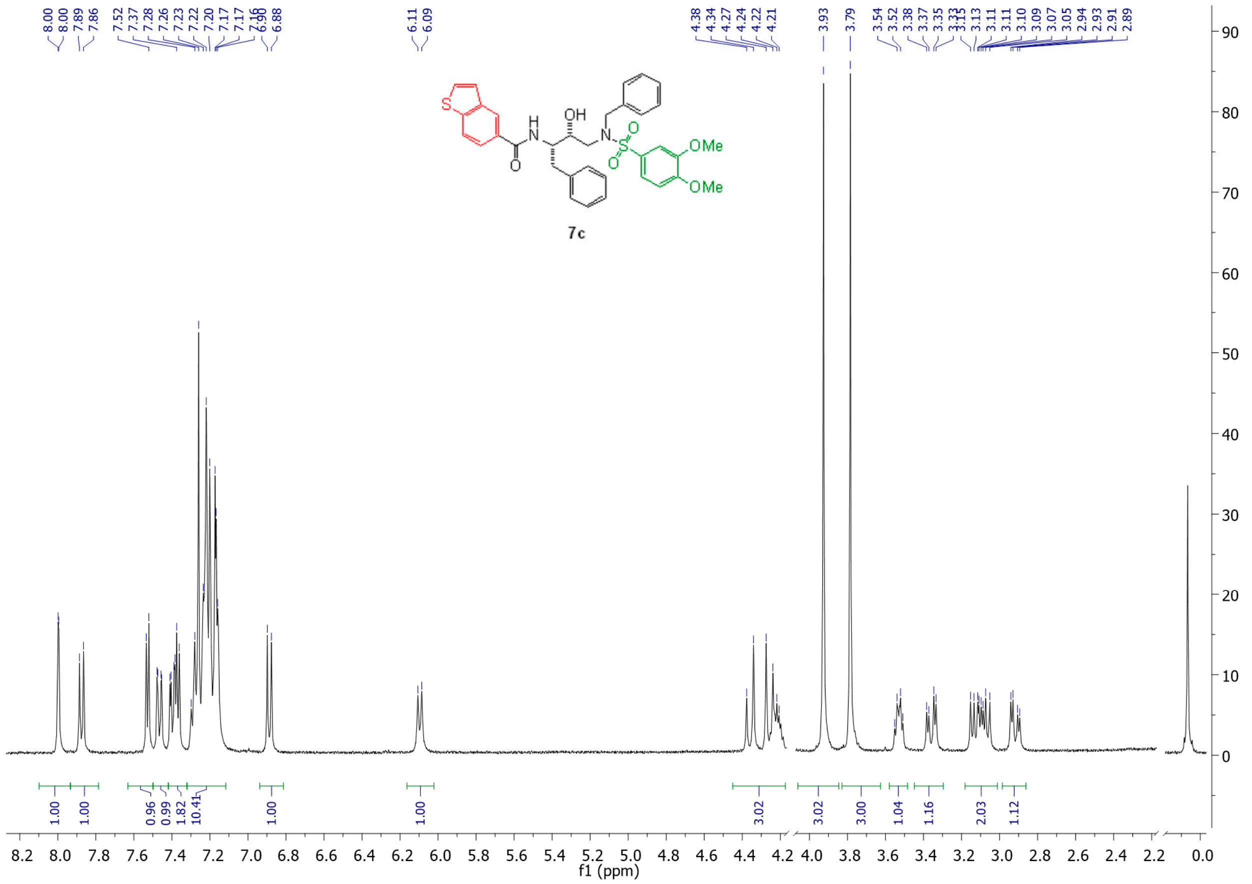
8.00
8.00
7.89
7.86
7.52
7.37
7.28
7.26
7.23
7.22
7.20
7.17
7.17
6.88
6.11
6.09
4.38
4.34
4.27
4.24
4.22
4.21
3.93
3.79
3.54
3.52
3.38
3.37
3.35
3.13
3.11
3.11
3.10
3.09
3.07
3.05
2.94
2.93
2.91
2.89
S
H
N
O
OH
N
S
O
O
OMe
OMe
7c
1.00
1.00
0.96
0.99
1.82
10.41
1.00
1.00
3.02
3.02
3.00
1.04
1.16
2.03
1.12
f1 (ppm)

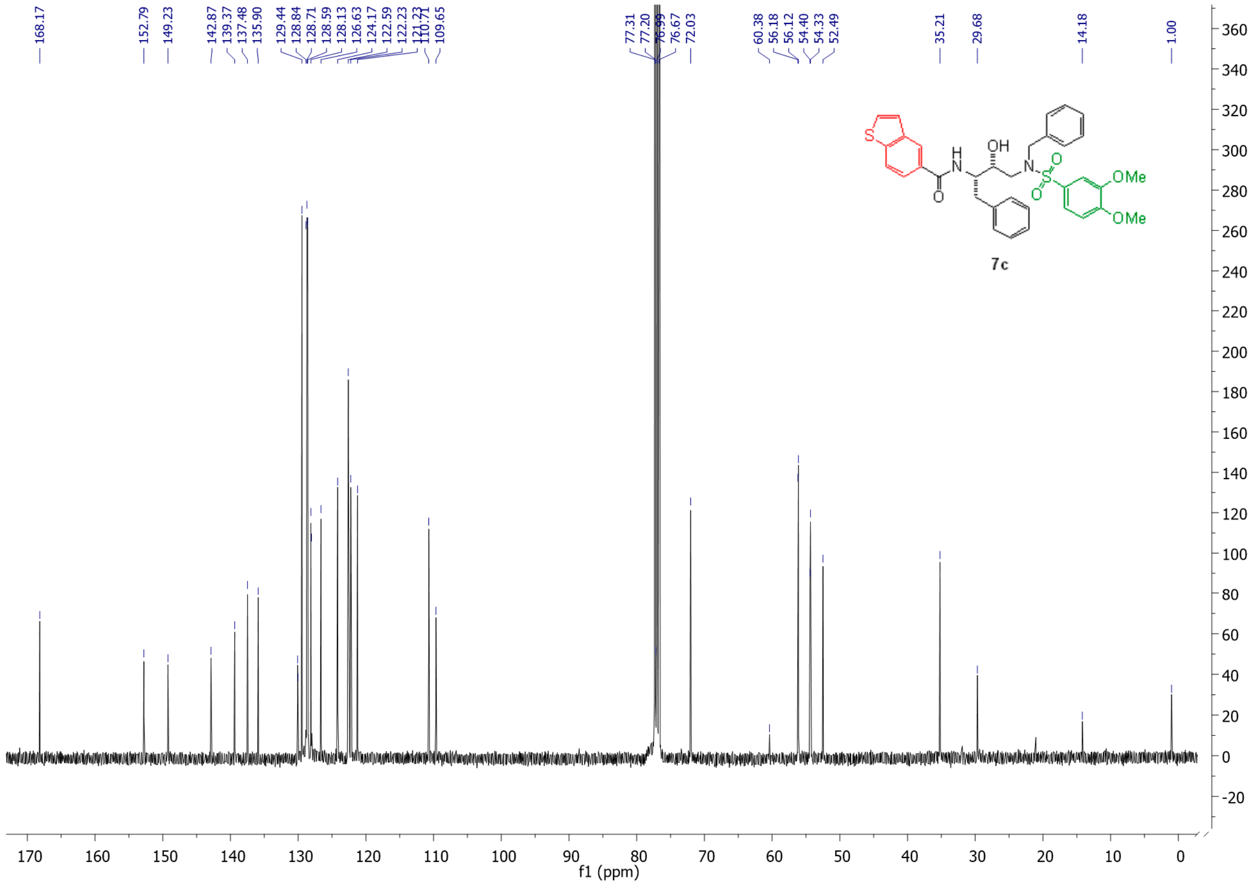
168.17
152.79
149.23
142.87
139.37
137.48
135.90
129.44
128.84
128.71
128.59
128.13
126.63
124.17
122.59
122.23
121.23
109.65
77.31
77.20
76.67
72.03
60.38
56.18
56.12
54.40
54.33
52.49
35.21
29.68
14.18
1.00
S
H
N
O
OH
N
S
O
O
OMe
OMe
7c
f1 (ppm)

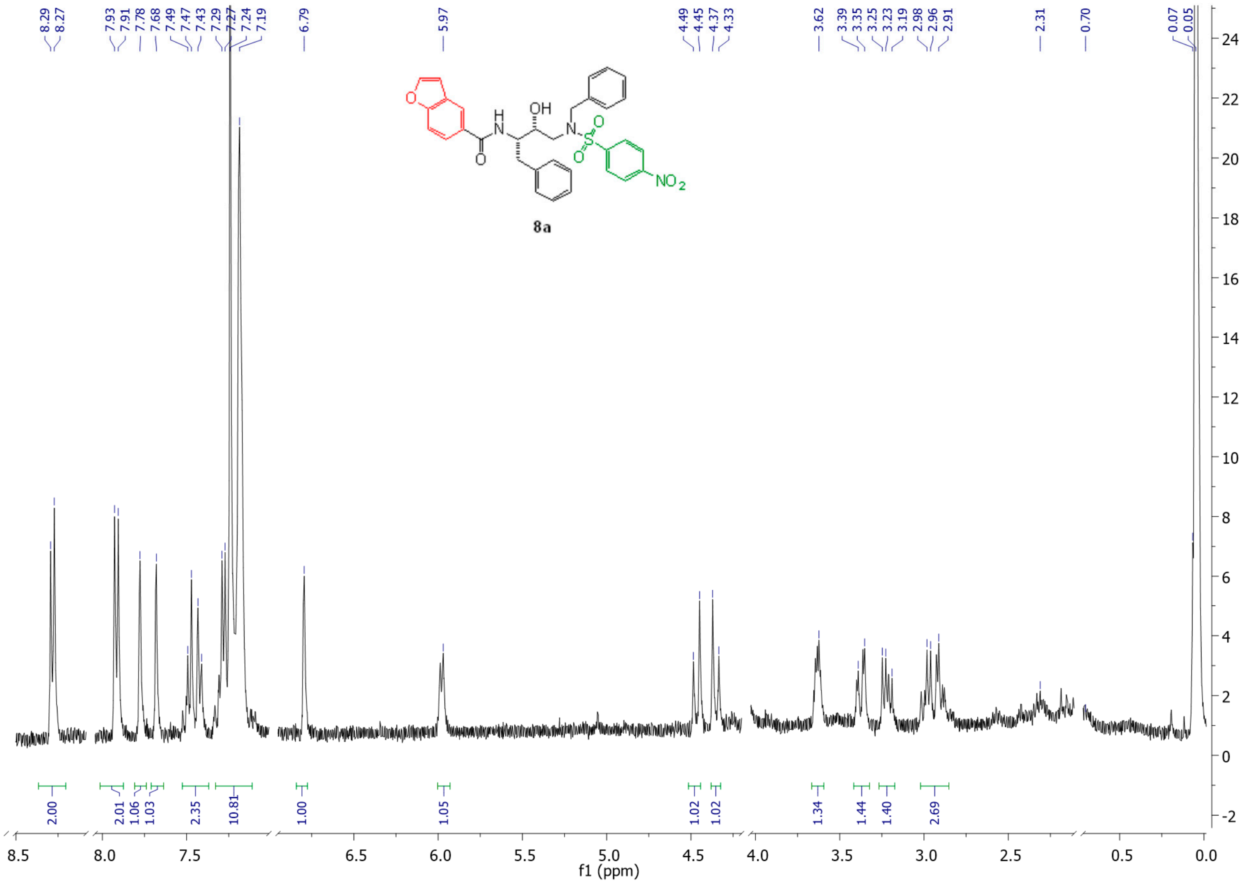
8.29
8.27
7.93
7.91
7.78
7.68
7.49
7.47
7.43
7.29
7.27
7.24
7.19
6.79
5.97
4.49
4.45
4.37
4.33
3.62
3.39
3.35
3.25
3.23
3.19
2.98
2.96
2.91
2.31
0.70
0.07
0.05
OH
H
N
O
O
S
O
NO2
8a
2.00
2.01
1.06
1.03
2.35
10.81
1.00
1.05
1.02
1.02
1.34
1.44
1.40
2.69
f1 (ppm)

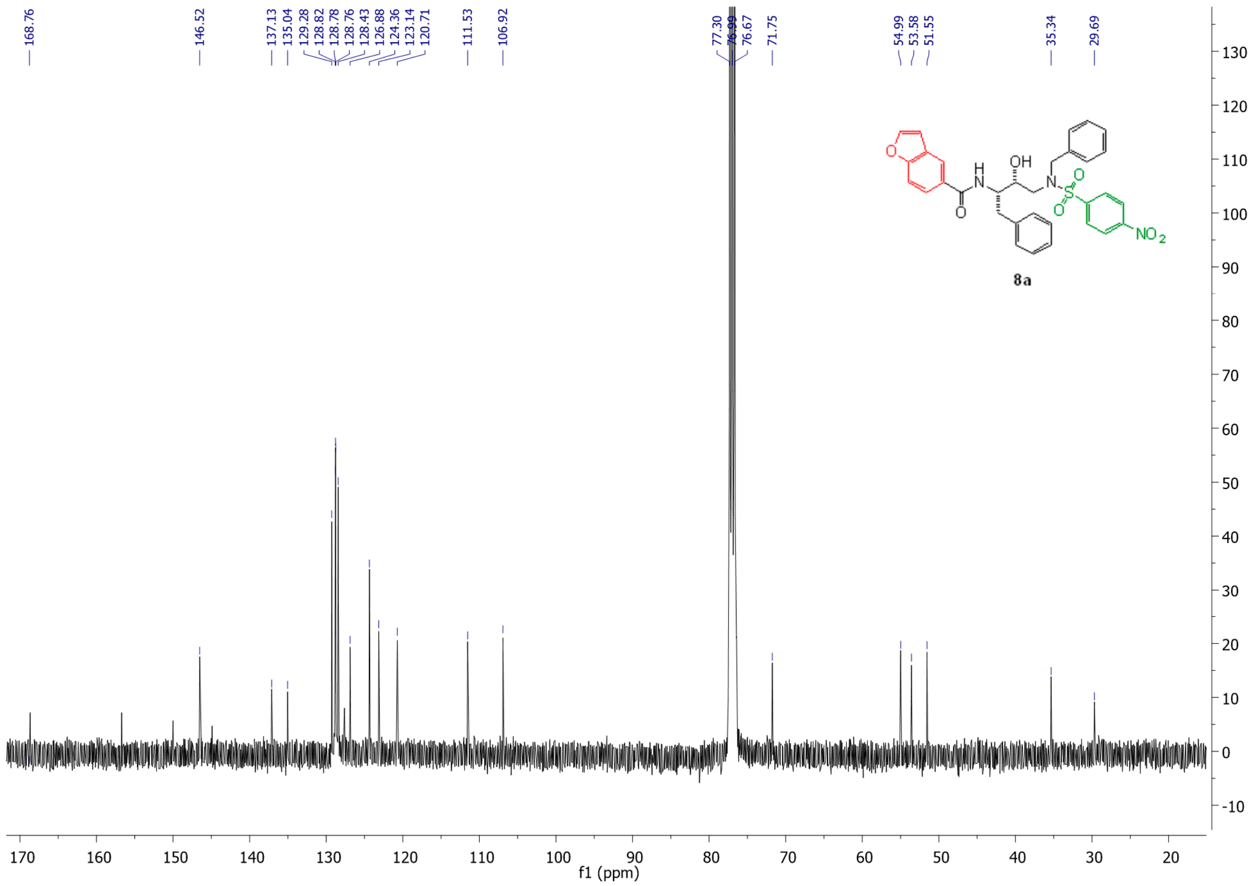
168.76
146.52
137.13
135.04
129.28
128.82
128.78
128.76
128.43
126.88
124.36
123.14
120.71
111.53
106.92
77.30
76.99
76.67
71.75
54.99
53.58
51.55
35.34
29.69
OH
H
N
O
O
S
O
NO2
8a
f1 (ppm)

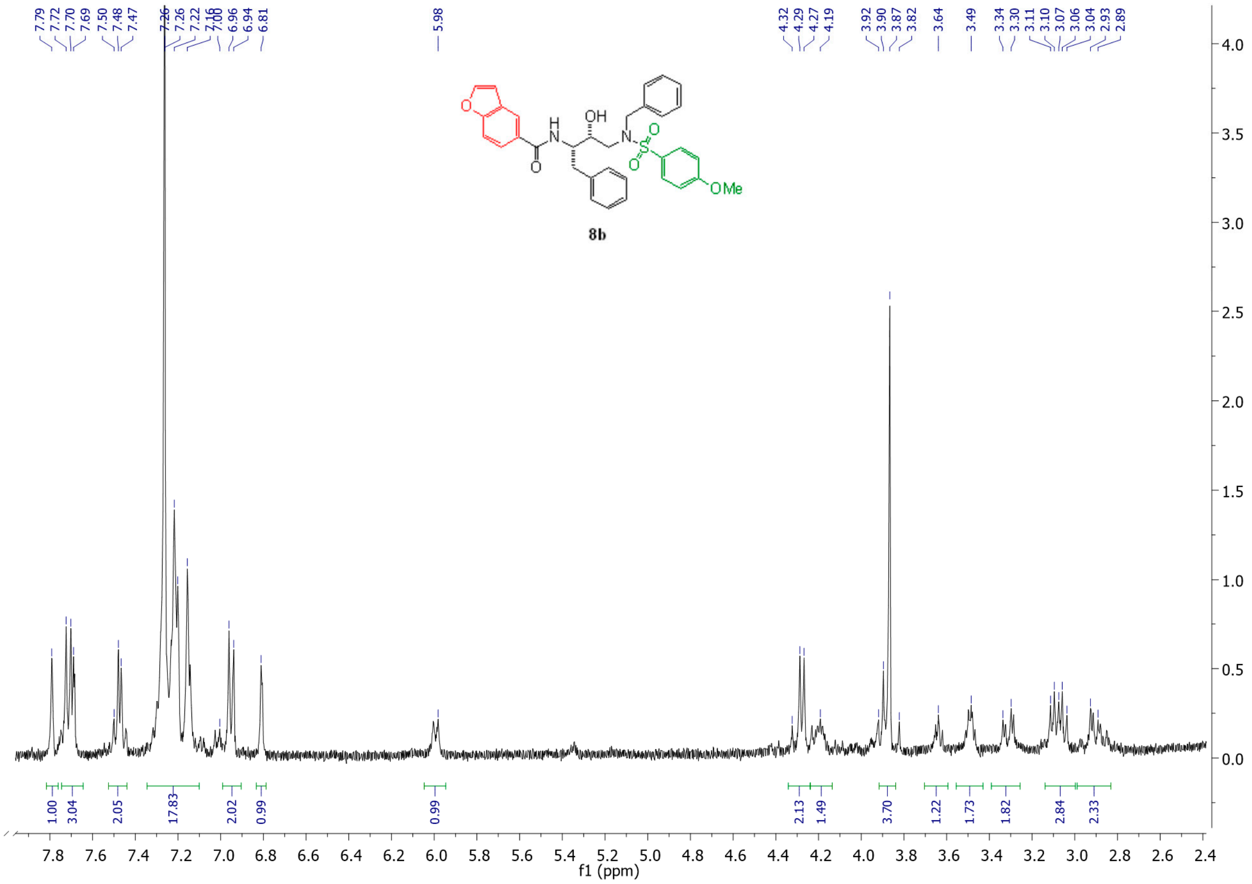
f1 (ppm)

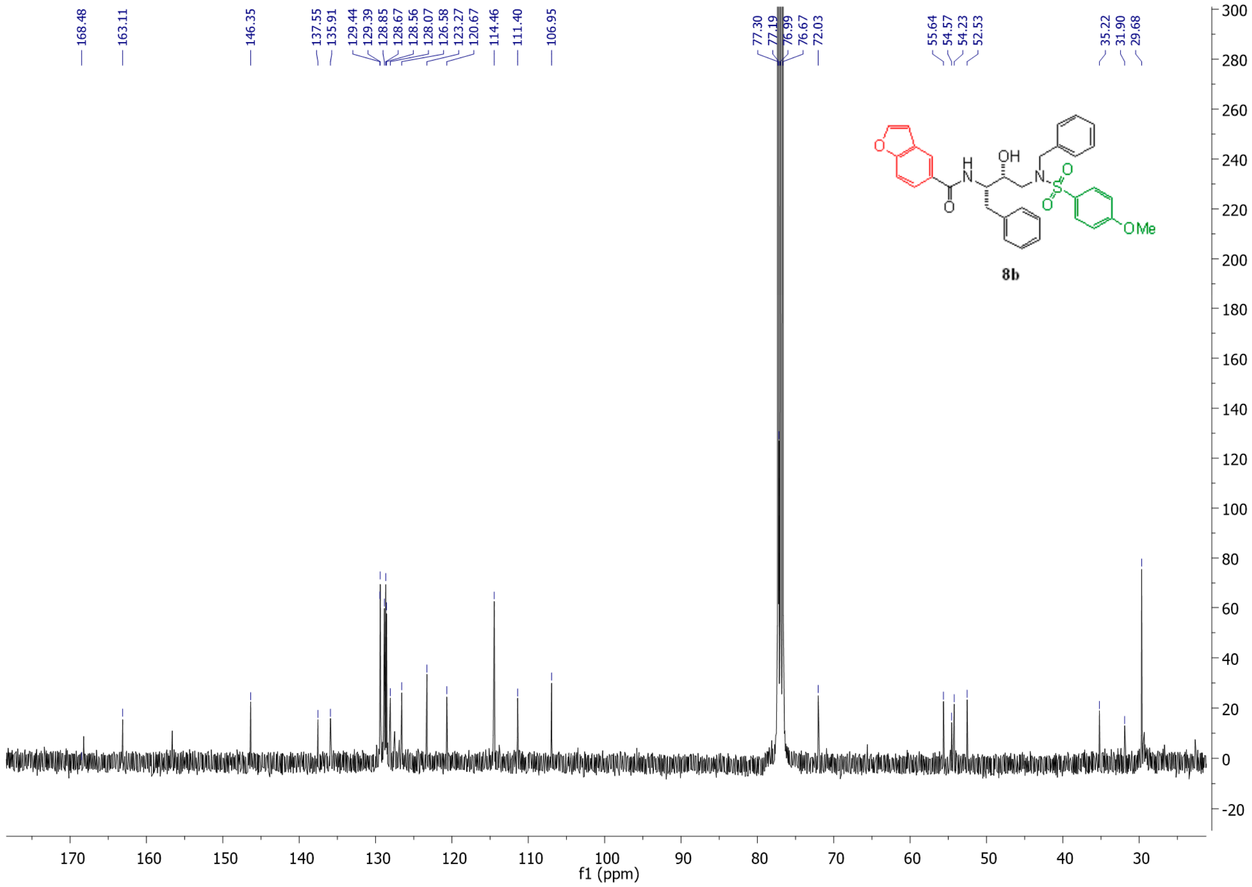
f1 (ppm)

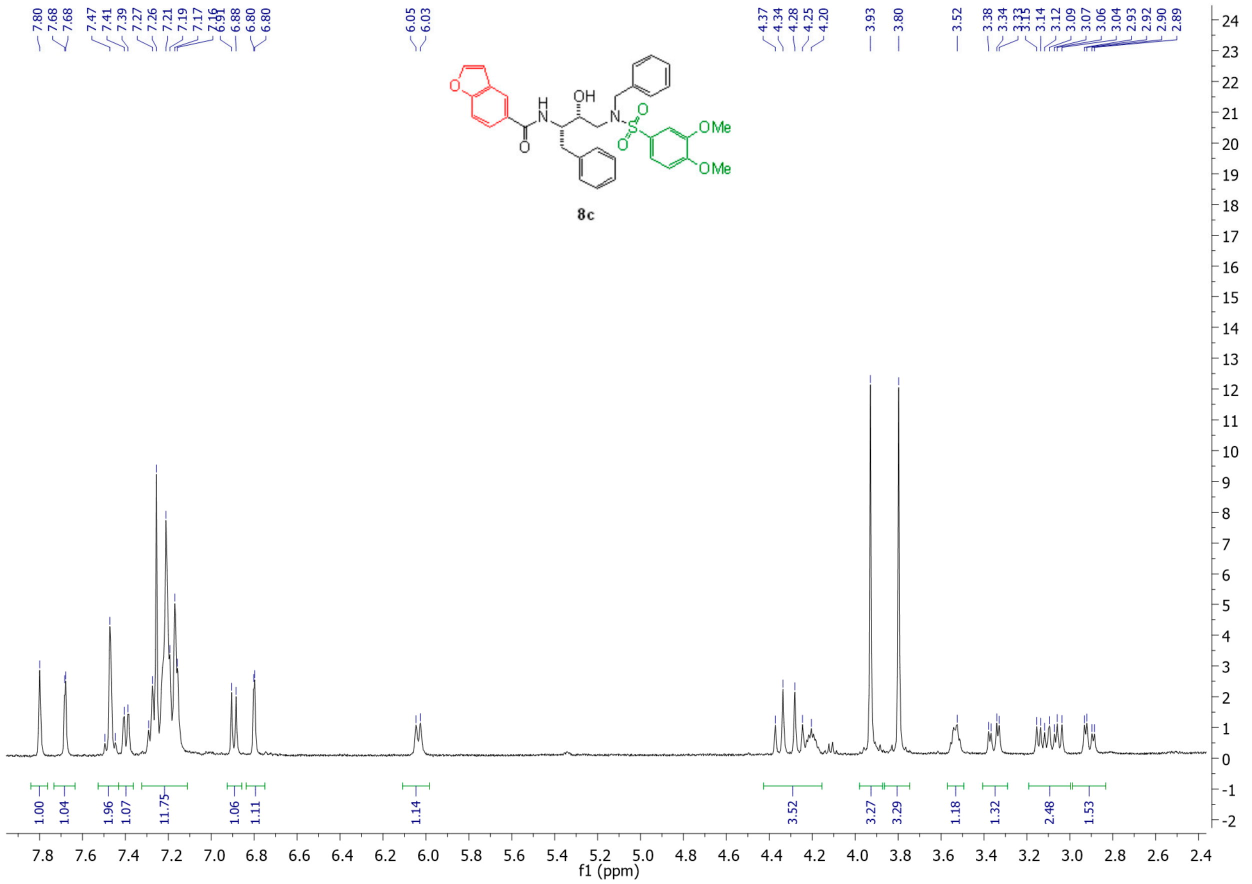

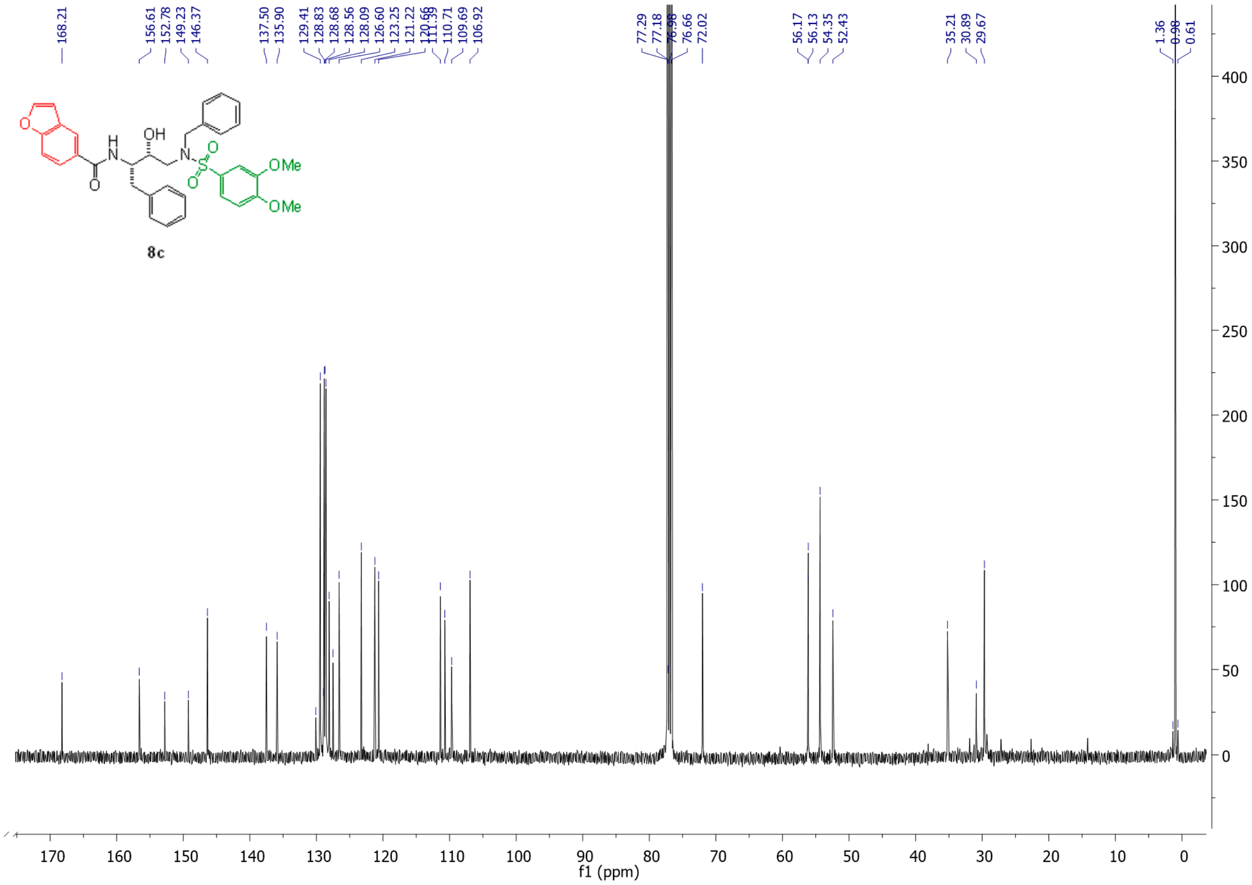

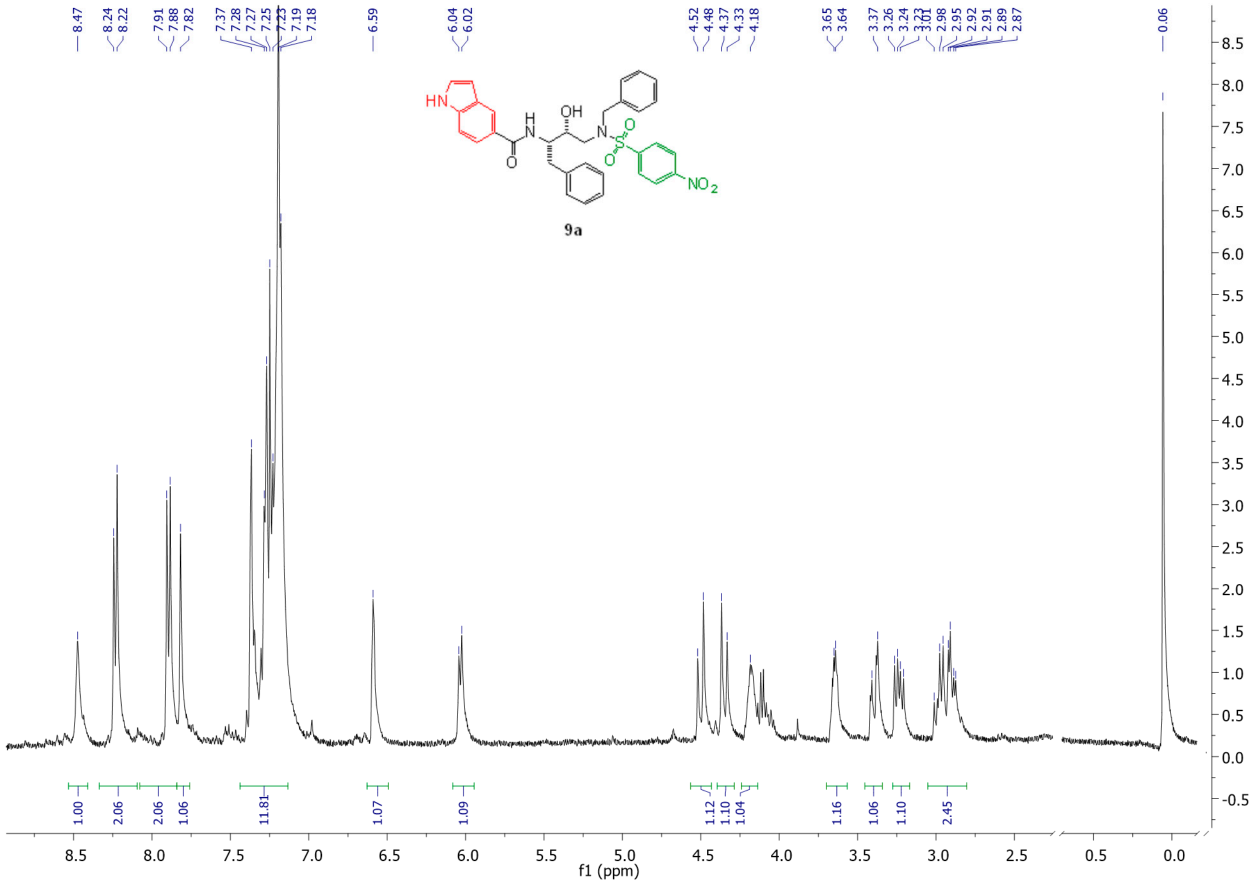
8.47
8.24
8.22
7.91
7.88
7.82
7.37
7.28
7.27
7.25
7.23
7.19
7.18
6.59
6.04
6.02
4.52
4.48
4.37
4.33
4.18
3.65
3.64
3.37
3.26
3.24
3.23
3.01
2.98
2.95
2.92
2.91
2.89
2.87
0.06
HN
H
N
OH
N
S
O
O
NO_2
9a
1.00
2.06
2.06
1.06
11.81
1.07
1.09
1.12
1.10
1.04
1.16
1.06
1.10
2.45
f1 (ppm)

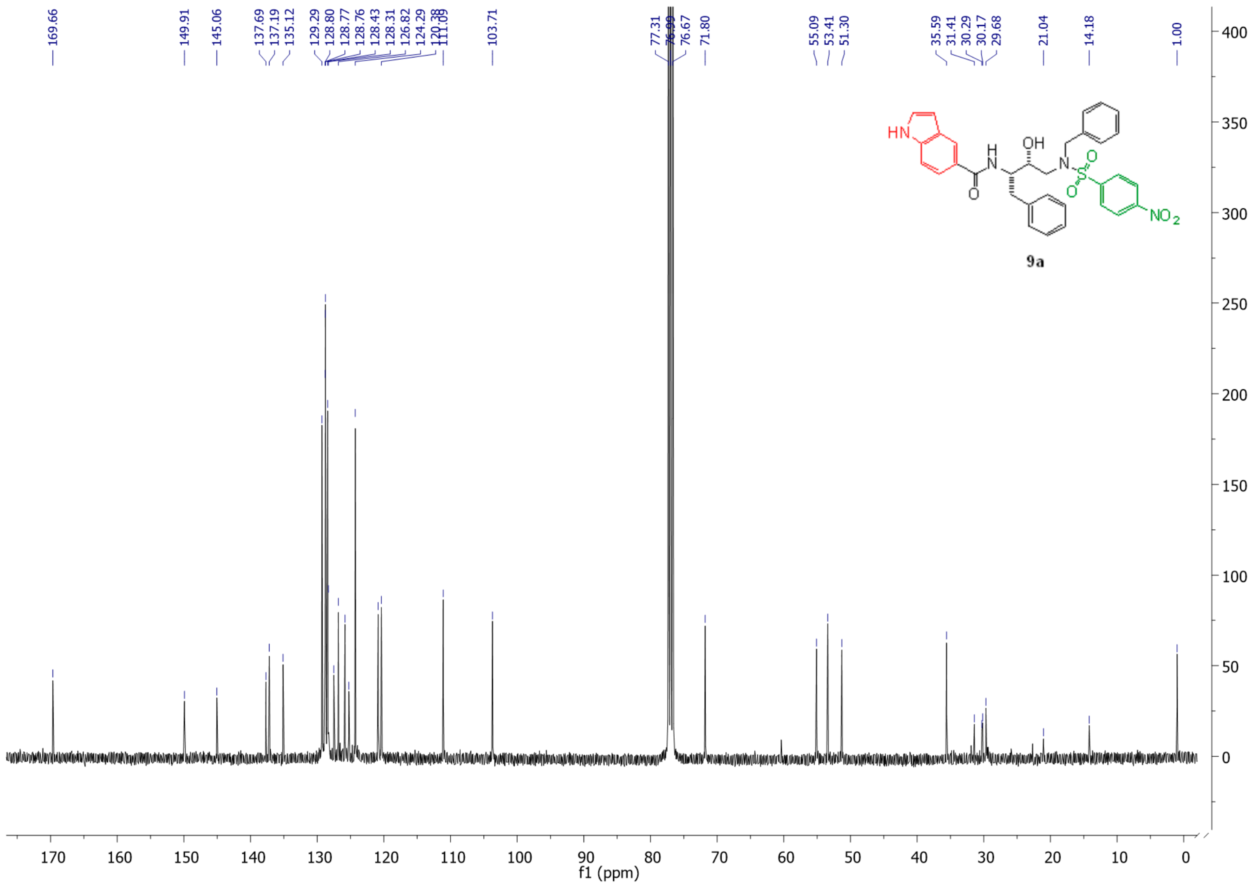
169.66
149.91
145.06
137.69
137.19
135.12
129.29
128.80
128.77
128.76
128.43
128.31
126.82
124.29
120.88
111.03
103.71
77.31
76.99
76.67
71.80
55.09
53.41
51.30
35.59
31.41
30.29
30.17
29.68
21.04
14.18
1.00
HN
H
N
OH
N
S
O
O
NO_2
9a
f1 (ppm)

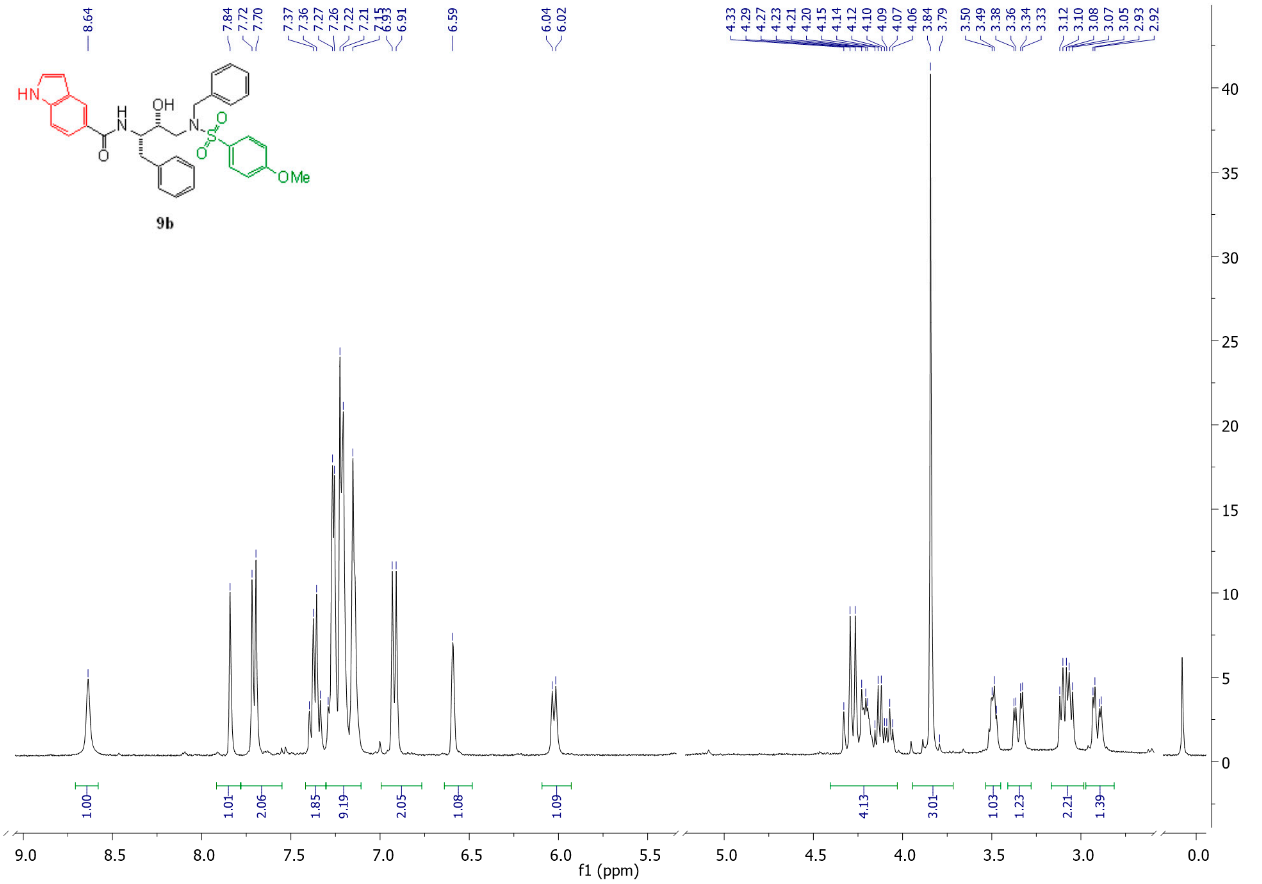
9b
f1 (ppm)

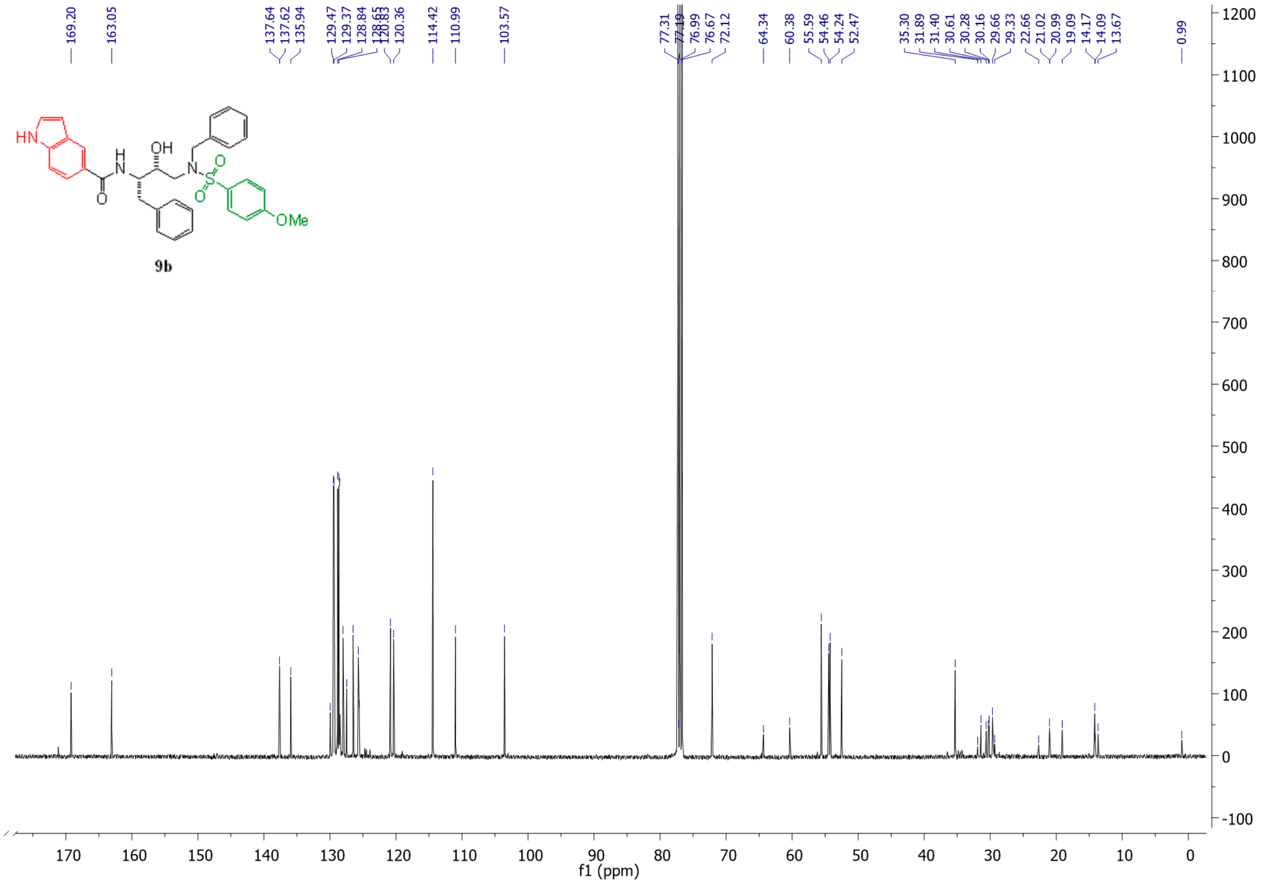
9b
f1 (ppm)

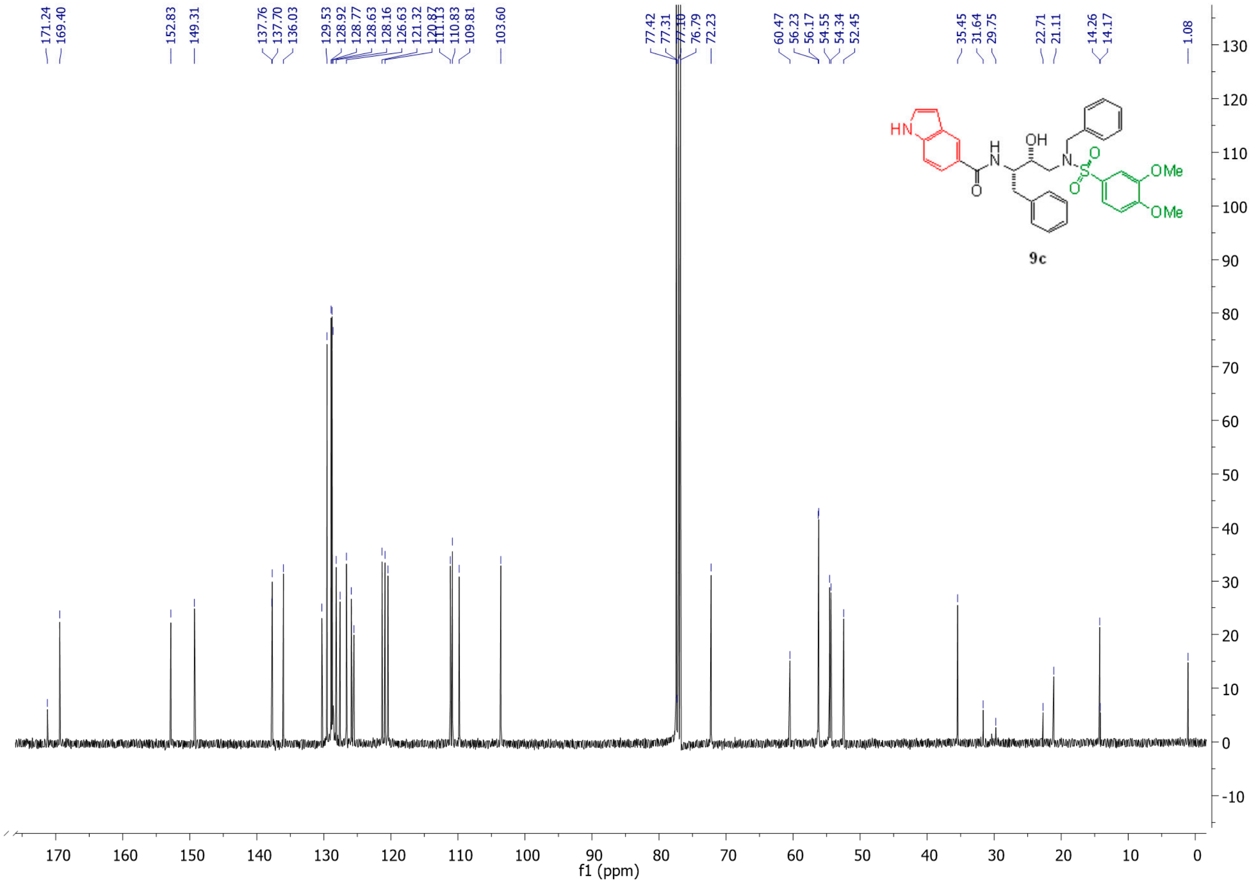
171.24
169.40
152.83
149.31
137.76
137.70
136.03
129.53
128.92
128.77
128.63
128.16
126.63
121.32
111.13
110.83
109.81
103.60
77.42
77.31
76.79
72.23
60.47
56.23
56.17
54.55
54.34
52.45
35.45
31.64
29.75
22.71
21.11
14.26
14.17
1.08
HN
H
N
OH
N
S
O
O
O
OMe
OMe
9c
f1 (ppm)

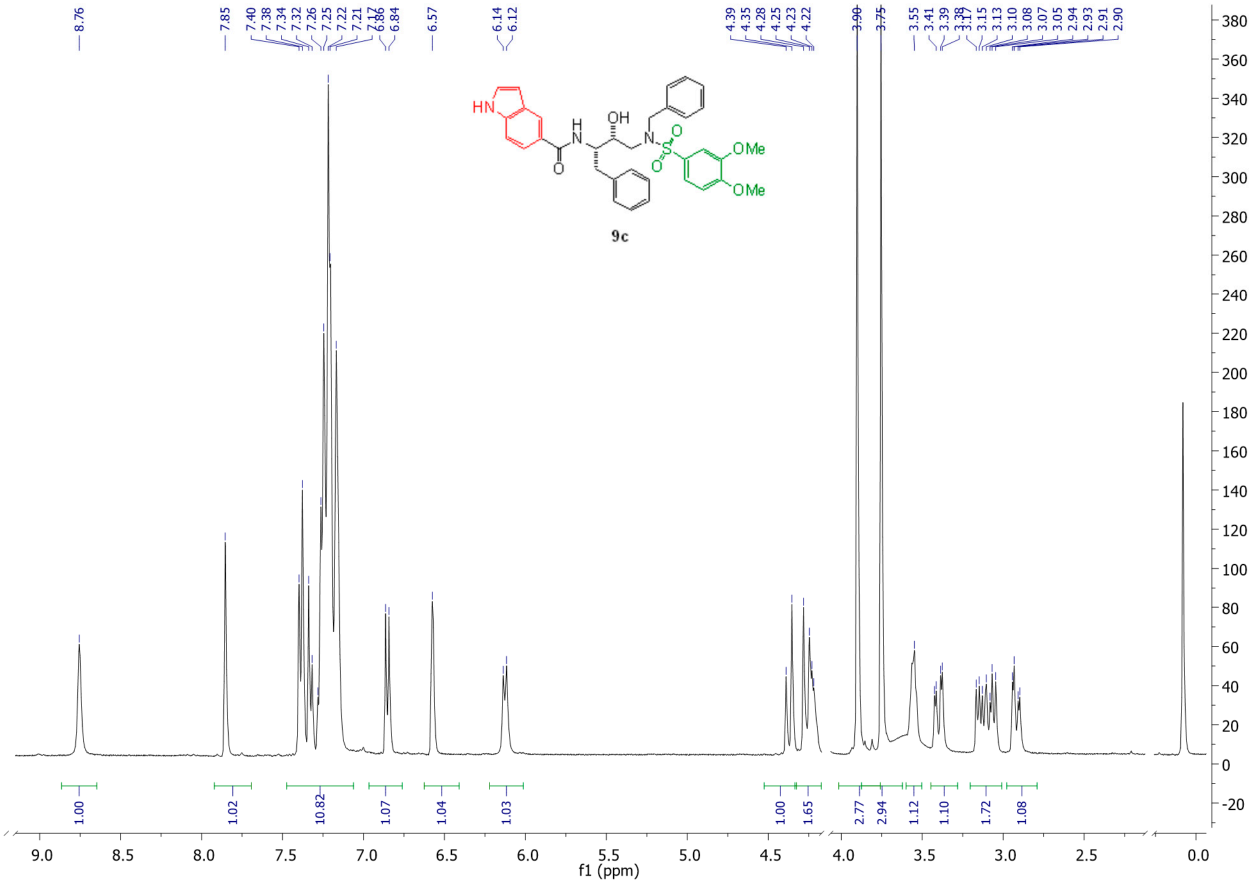
8.76
7.85
7.40
7.38
7.34
7.32
7.26
7.25
7.22
7.21
6.86
6.84
6.57
6.14
6.12
4.39
4.35
4.28
4.25
4.23
4.22
3.90
3.75
3.55
3.41
3.39
3.15
3.13
3.10
3.08
3.07
3.05
2.94
2.93
2.91
2.90
HN
H
N
OH
N
S
O
O
O
OMe
OMe
9c
1.00
1.02
10.82
1.07
1.04
1.03
1.00
1.65
2.77
2.94
1.12
1.10
1.72
1.08
f1 (ppm)

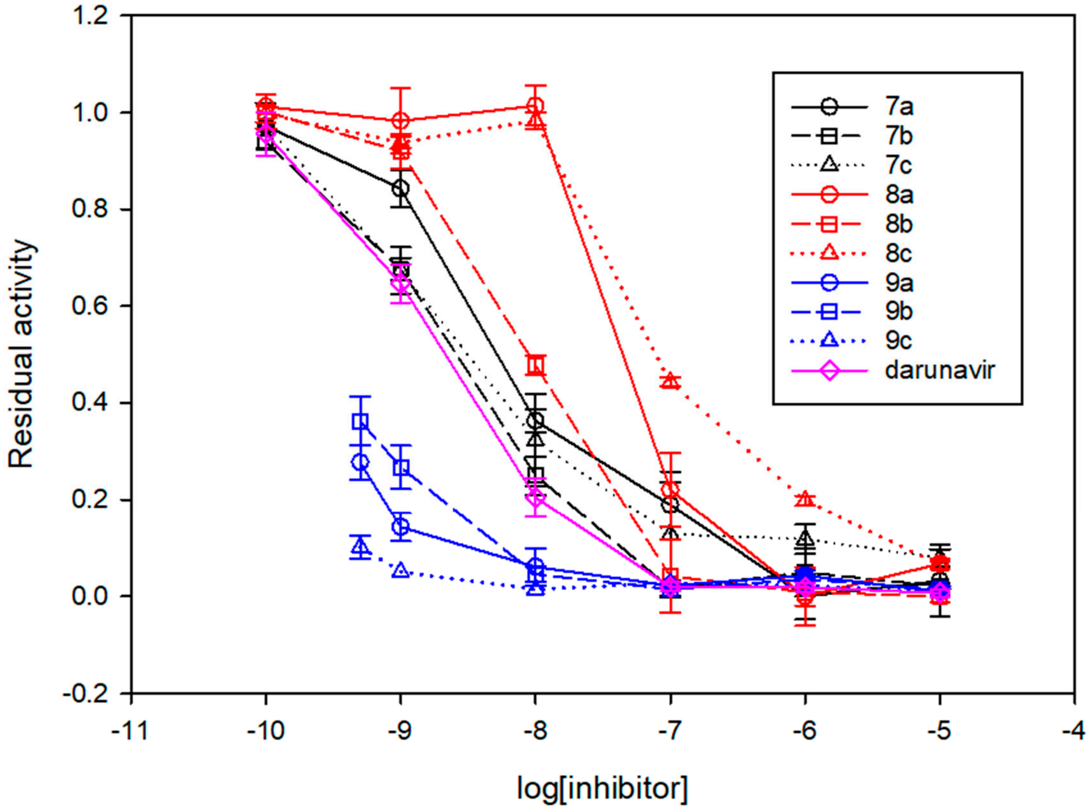

Supplementary Figure S1. concentration – response curves for the inhibition of the protease with all the inhibitors.

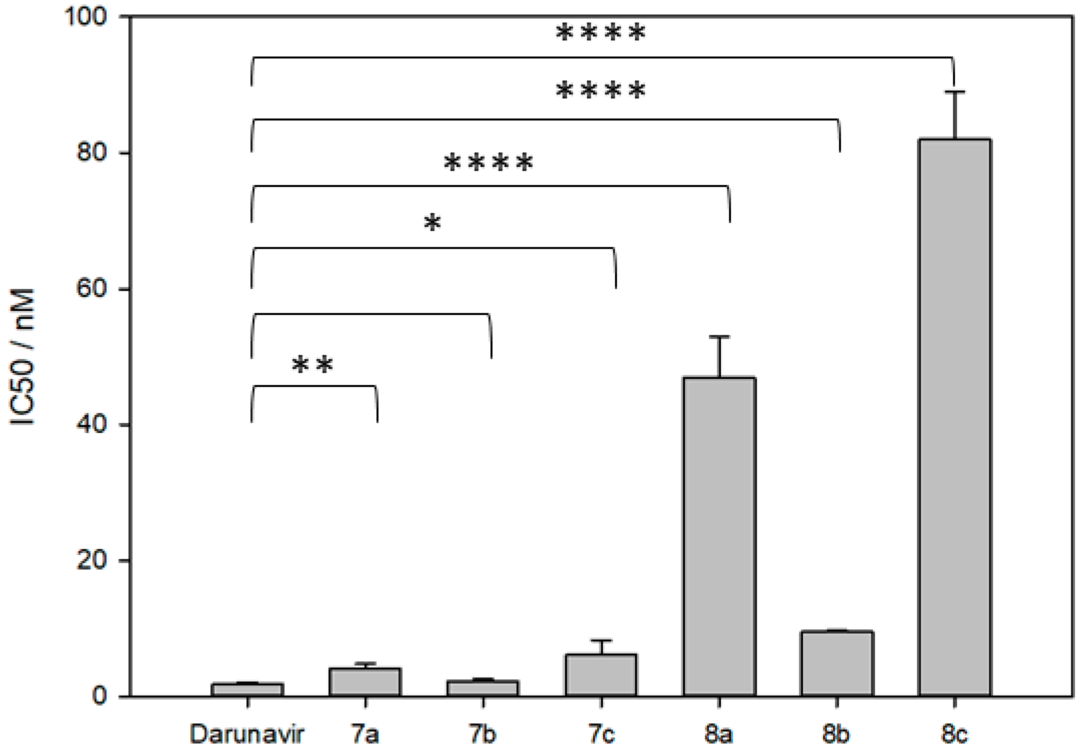

Supplementary Figure S2. statistical comparison of the measured IC50 with respect to darunavir. *: p<0.01; **: p<0.001; ****: p<0.00001.

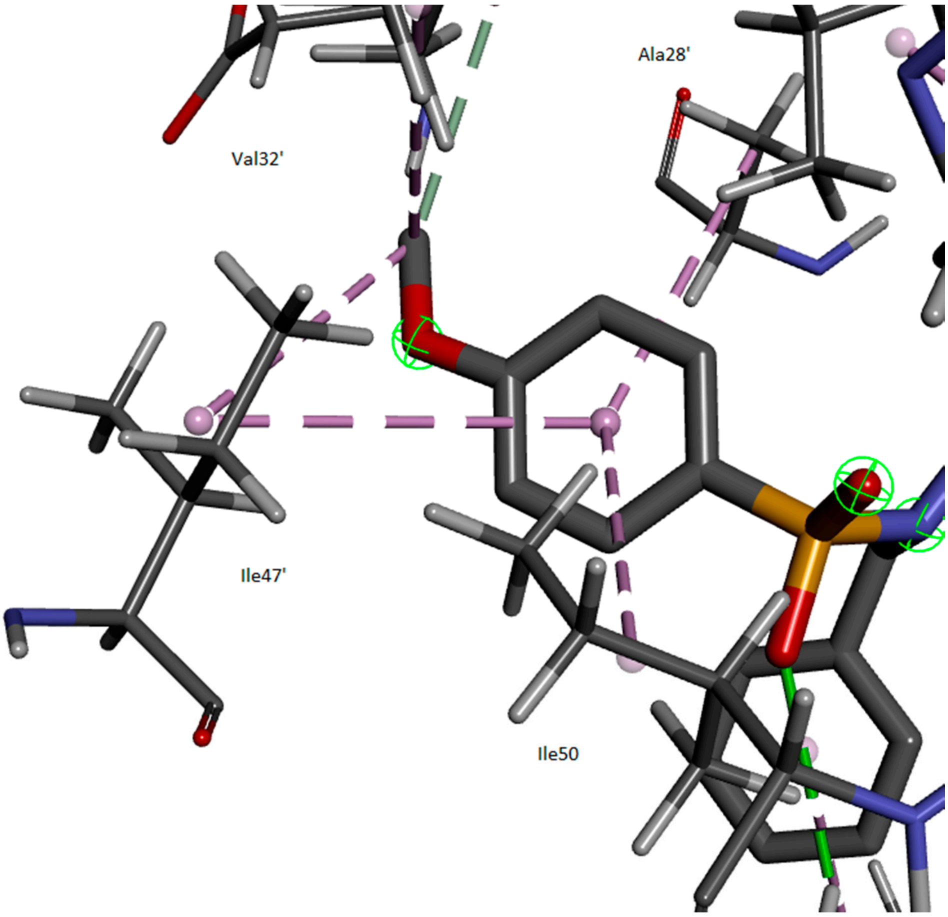

Supplementary figure S3: interactions of the 4-methoxyphenyl group at P2' in the model structure of the complex of compound **9b** with the protease.

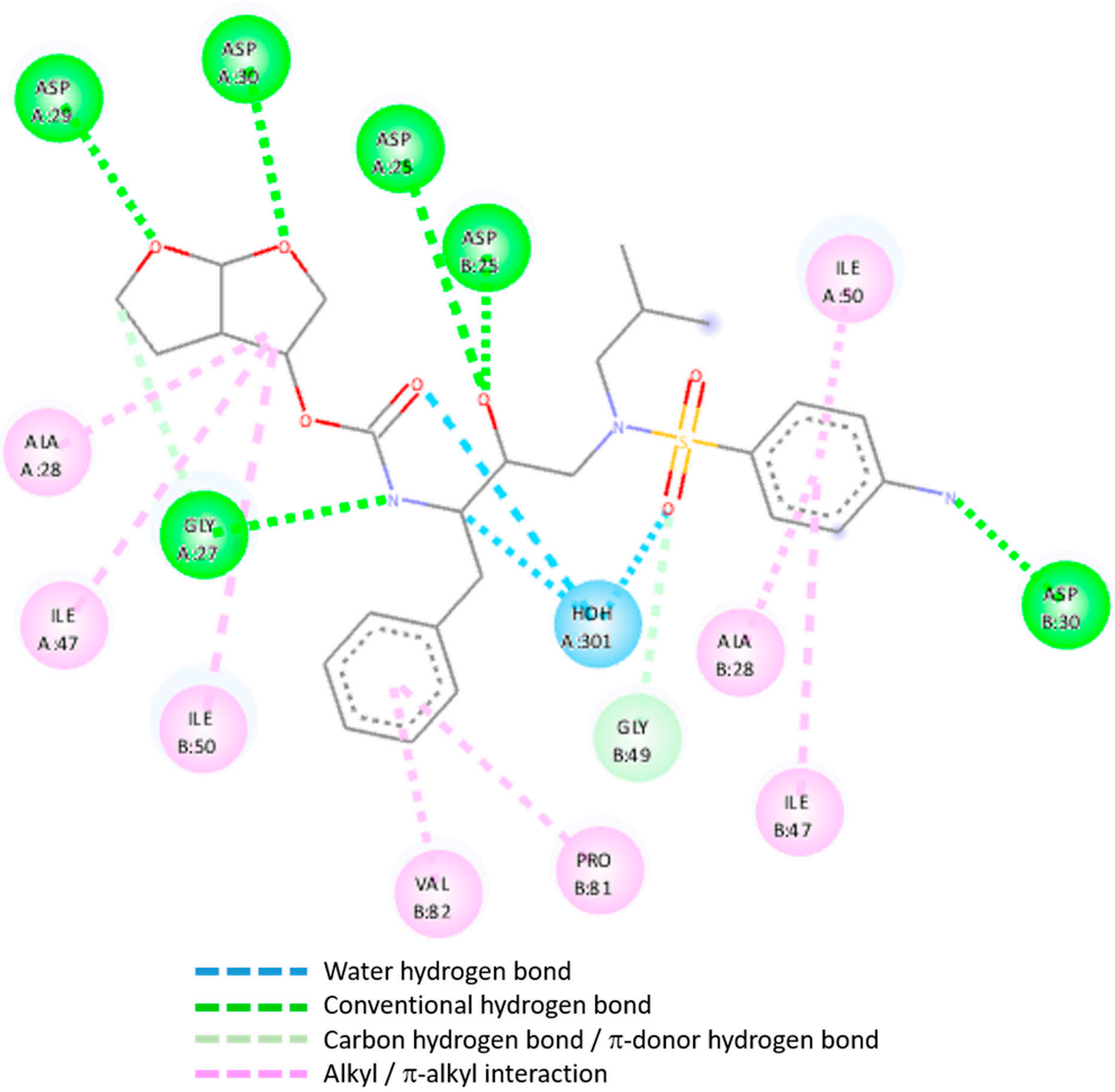

Supplementary figure S4: map of the interactions of Darunavir with the protease.

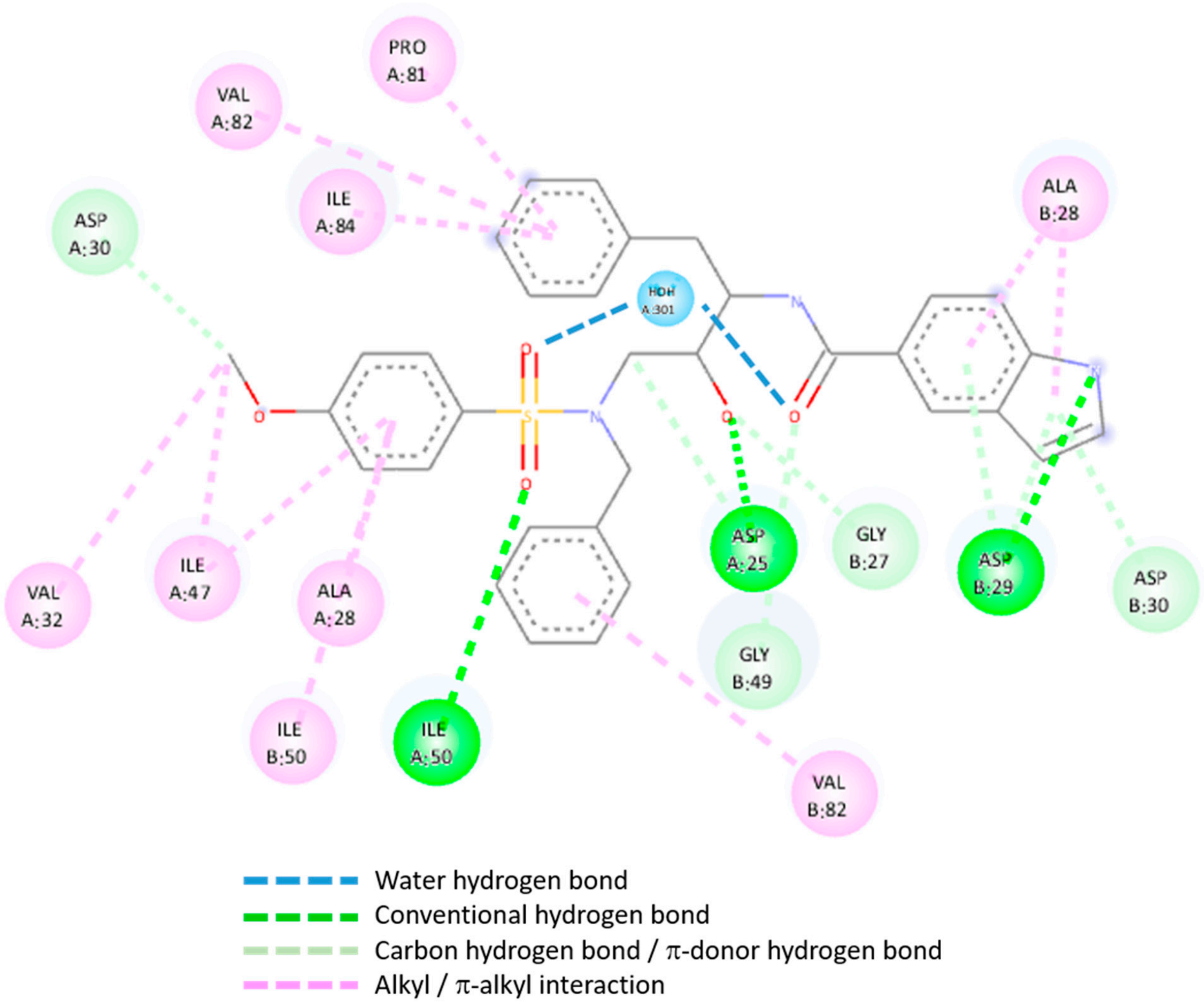

Supplementary figure S5: map of the interactions of compound **9b** with the protease.
